# Accurate digital quantification of tau pathology in progressive supranuclear palsy

**DOI:** 10.1186/s40478-023-01674-y

**Published:** 2023-11-09

**Authors:** Tanrada Pansuwan, Annelies Quaegebeur, Sanne S. Kaalund, Eric Hidari, Mayen Briggs, James B. Rowe, Timothy Rittman

**Affiliations:** 1 Department of Clinical Neurosciences, Cambridge University Centre for Parkinson-Plus, University of Cambridge, Herchel Smith Building, Robinson Way, Cambridge, CB2 0SZ UK; 2 Centre for Neuroscience and Stereology, Bispebjerg University Hospital, Copenhagen, Denmark; 3 Cambridge University Hospitals NHS Foundation Trust, Cambridge, UK; 4 Medical Research Council Cognition and Brain Sciences Unit, University of Cambridge, Cambridge, UK

**Keywords:** Digital pathology, Random forest, Neurodegenerative diseases, Machine learning, PSP

## Abstract

The development of novel treatments for Progressive Supranuclear Palsy (PSP) is hindered by a knowledge gap of the impact of neurodegenerative neuropathology on brain structure and function. The current standard practice for measuring *postmortem* tau histology is semi-quantitative assessment, which is prone to inter-rater variability, time-consuming and difficult to scale. We developed and optimized a tau aggregate type-specific quantification pipeline for cortical and subcortical regions, in human brain donors with PSP. We quantified 4 tau objects (‘neurofibrillary tangles’, ‘coiled bodies’, ‘tufted astrocytes’, and ‘tau fragments’) using a probabilistic random forest machine learning classifier. The tau pipeline achieved high classification performance (F1-score > 0.90), comparable to neuropathologist inter-rater reliability in the held-out test set. Using 240 AT8 slides from 32 *postmortem* brains, the tau burden was correlated against the PSP pathology staging scheme using Spearman’s rank correlation. We assessed whether clinical severity (PSP rating scale, PSPRS) score reflects neuropathological severity inferred from PSP stage and tau burden using Bayesian linear mixed regression. Tufted astrocyte density in cortical regions and coiled body density in subcortical regions showed the highest correlation to PSP stage (r = 0.62 and r = 0.38, respectively). Using traditional manual staging, only PSP patients in stage 6, not earlier stages, had significantly higher clinical severity than stage 2. Cortical tau density and neurofibrillary tangle density in subcortical regions correlated with clinical severity. Overall, our data indicate the potential for highly accurate digital tau aggregate type-specific quantification for neurodegenerative tauopathies; and the importance of studying tau aggregate type-specific burden in different brain regions as opposed to overall tau, to gain insights into the pathogenesis and progression of tauopathies.

**Supplementary Information:**

The online version contains supplementary material available at 10.1186/s40478-023-01674-y.

## Introduction

Many neurodegenerative diseases are characterised by abnormal protein accumulation within neurons and glia [1, 2]. Understanding the severity and distribution of this protein pathology is key to investigate the aetiology, understand disease heterogeneity, model disease progression, and to design molecular-targeted disease-modifying therapies. Hyperphosphorylated and misfolded aggregates of tau accumulate in common and rare neurodegenerative diseases, including Alzheimer’s disease, frontotemporal dementia, and Progressive Supranuclear Palsy (PSP). Such tau pathology is related to neuronal loss [3], grey matter atrophy [4] and clinical severity [5, 6].

Here we focus on PSP, a primary tauopathy [1] characterised by the accumulation of 4-repeat tau in neuronal and glial cells, without the accumulation of beta-amyloid, as seen in Alzheimer’s disease, or alpha-synuclein, as seen in Parkinson’s disease. The typical Richardson Syndrome of PSP includes vertical gaze palsy, falls, dysarthria, dysphagia, and cognitive impairment [7]. In PSP, tau forms distinct and recognisable features in different cell types, including tufted astrocytes, coiled bodies in oligodendrocytes, and neurofibrillary tangles and threads in neurons. These features support a staging scheme for the progression of PSP pathology based on *postmortem* analysis [2, 8]. However, the current standard practice for measuring the density of tau pathology is semi-quantitative where pathologists visually grade the severity of pathology on a simple ordinal scale [9]. This standard manual assessment has limitations. It requires extensive training to accurately identify different morphologies of tau aggregates and cell types [38]. It is slow, or limited to small set of sub-regions, and it is subjective due to innate differences in visual perception and decision-making processes between individuals, even amongst equally trained pathologists [10, 11]. High-throughput, reliable, automated methods capable of comprehensive coverage could address these limitations.

Machine learning approaches have been applied to move towards more objective and scalable solutions for digital pathology [12–14]. However, quantitative pathology with machine learning has many challenges to properly assess validation, interpretability, and standardisation [15]. If these can be addressed, machine learning approaches have the potential to address the need for more sensitive measures of disease burden [9, 16]. One class of machine learning models used in biomedical and bioinformatic research are probabilistic classifiers, which include random forest classifiers. Advantages of random forest algorithms are that they are relatively simple to train and cope well with imbalanced datasets [17, 18]. This makes them particularly suitable for classifying neuropathology since the proportion of neuronal and glial cells in the brain, and therefore types of tau aggregates, is imbalanced [19–22].

In the present study, we aimed to quantify tau pathology in PSP *postmortem* brains by developing a digital tau pathology pipeline for whole slide images using a random forest algorithm. This pipeline has been developed to work with brain regions included in the current consensus PSP pathology staging scheme [2] and additional cortical regions relevant to PSP. There are 3 main methodological challenges in tau classification across multiple brain regions that we have tried to address. First, there are not equal numbers of neuronal and glial cells in the brain, leading to a class imbalance for the machine learning model. Second, the ratio of class imbalance and tau morphology differs between brain regions. Third, there is inherent ambiguity in classifying some tau objects, even for expert neuropathologists. We therefore designed our pipeline with these challenges in mind. The random forest algorithm inherently manages class imbalance, and by developing classifiers specific for four different groups of brain regions, we were able to optimise the classifier for class imbalance between regions. Finally, we explicitly addressed the challenge of ambiguous classification by optimising thresholds for each class of tau object and excluding individual objects that met either no class threshold or multiple class thresholds.

We applied the optimised algorithm to quantify tau pathological hallmarks of PSP which include ‘coiled bodies’ (CB), ‘neurofibrillary tangles’ (NFT), ‘tufted astrocytes’ (TA) and ‘tau fragments’ (TF). We use the resulting estimates of regional tau pathology to test the relationship between quantified PSP stage and clinical severity.

## Materials and methods

### Donors and brain regions

A total of 240 formalin-fixed paraffin embedded slides were obtained from 32 brains (2–10 slides per brain, median = 8.5, IQR = 6) donated by patients with a clinical and pathological diagnosis of progressive supranuclear palsy (PSP) that also meet Rainwater criteria of PSP (Table 1) to the Cambridge Brain Bank under the Neuropathology Research in Dementia (NERD) study with ethical approval from the Wales 6 Research Ethics Committee. The slides included 185 cortical slides (29 pre-frontal, 21 premotor, 20 primary motor, 22 primary somatosensory, 23 temporal, 20 parietal, 28 occipital, 22 cingulate), 25 basal ganglia and 30 cerebellar (dentate nucleus) slides. Of the 240 slides, 13 slides were used for model development and 6 as a held-out test set. Training and held-out test slides were annotated by a trained expert (TP), and a neuropathologist (AQ) independently annotated the held-out test slides to calculate the inter-rater reliability. Following pipeline development, 227 novel slides were used for validation against the PSP staging scheme [2] and all slides were used for further analyses.

**Table 1:** Clinical and neuropathological data of donor participants with pathological diagnosis of PSP in the study

Subject	Age at death (years)	Gender	Clinical diagnosis	Disease duration (years)	Last PSPRS Total	PSPRS to death (years)	Pathological stage	GP	STN	STR	PF	DN	OC
1	76	Female	prob. PSP-RS	8.75	63	0.32	2	2	2	2	0	1	0
2	75	Male	poss. PSP-PGF	4.62	26	0.52	2	3	3	2	0	1	0
3	55	Female	prob. PSP-RS	5.5	53	0.41	3	2	2	2	1	1	0
4	74	Male	prob. PSP-RS	6	45	0.67	3	2	2	2	1	1	0
5	72	Male	prob. PSP-RS	N/A	N/A	N/A	3	N/A	N/A	N/A	N/A	N/A	N/A
6	65	Female	prob. PSP-RS	13.92	54	0.75	4	2	3	2	1	N/A	0
7	78	Male	poss. PSP-CBS	5.75	43	0.69	4	3	3	3	3	2	0
8	79	Female	poss. PSP-CBS	3.42	49	0.74	4	2	2	2	1	2	0
9	77	Male	prob. PSP-RS	6.33	55	0.74	4	3	3	2	2	3	0
10	78	Male	prob. PSP-RS	5.33	54	0.56	4	2	2	2	1	2	0
11	80	Male	prob. PSP-RS	6.5	62	0.11	4	2	3	2	1	2	0
12	71	Female	prob. PSP-RS	4.58	45	0.13	4	2	3	3	1	2	0
13	75	Female	poss. PSP-CBS	2.83	N/A	N/A	4	3	N/A	2	1	2	0
14	64	Male	prob. PSP-RS	5.08	38	1.09	4	2	N/A	2	2	3	0
15	80	Male	prob. PSP-RS	11.92	76	1.3	4	2	3	2	1	2	0
16	71	Male	prob. PSP-RS	5.17	38	2.21	5	2	3	2	3	2	1
17	78	Female	poss. PSP-SL	8.83	72	1.94	5	3	3	3	3	3	1
18	63	Male	prob. PSP-RS	8.83	62	2.44	5	3	3	3	2	3	1
19	76	Male	prob. PSP-RS	3.87	51	0.42	5	3	3	3	3	3	1
20	74	Female	prob. PSP-RS	6.58	58	2.66	5	2	2	2	2	2	1
21	88	Male	prob. PSP-RS	5.08	53	0.3	5	2	N/A	2	2	2	1
22	69	Female	prob. PSP-RS	5.33	51	0.36	5	3	3	3	2	3	1
23	71	Female	prob. PSP-RS	6.17	60	0.05	5	2	N/A	2	2	3	1
24	71	Male	prob. PSP-RS	5.42	43	1.89	5	3	3	3	3	3	1
25	73	Male	poss. PSP-CBS	4	NA	NA	5	3	3	3	3	3	1
26	78	Female	poss. PSP-CBS	5	48	0.18	5	3	3	3	1	3	1
27	84	Male	prob. PSP-RS	8.75	59	2.42	5	3	3	3	2	3	1
28	84	Female	prob. PSP-RS	4.25	67	0.78	5	3	3	3	3	3	1
29	78	Female	prob. PSP-RS	16.75	52	0.99	5	3	3	3	3	2	1
30	78	Male	poss. PSP-CBS	N/A	N/A	N/A	5	N/A	N/A	N/A	N/A	N/A	N/A
31	80	Female	poss. PSP-CBS	8.42	73	0.23	6	3	3	2	2	3	2
32	75	Male	prob. PSP-F	8.42	81	0.76	6	3	3	3	3	3	2

Not applicable (N/A) where data is not available in the database. Severity rating for each brain region includes 0 = absence, 1 = mild, 2 = moderate, 3 = severe. For clinical diagnosis of PSP participants, *prob.* probable, *poss.* possible, s.o. suggestive of, *RS* Richardson syndrome, *CBS* predominant corticobasal syndrome, *SL* predominant speech and language disorder, *F* predominant frontal presentation, *PGF* progressive gait freezing. *PSPRS* PSP rating scale (clinical severity measure). *GP* Globus pallidus, *STN* Subthalamic nucleus, *STR* Striatum, *PF* pre-frontal, *DN* Dentate nucleus, *OC* Occipital

### Tissue processing and immunohistochemistry

Immunostaining for hyperphosphorylated tau using AT8 (MN1020, Thermo Scientific, USA) was performed, followed by 3,3′-diaminobenzidine (DAB) staining to visualise pathological tau as a brown reaction product. Counter-staining was performed using haematoxylin to visualise cell nuclei as blue reaction products. Slide images were acquired by an Aperio AT2 whole slide scanner (Leica) at 40× magnification.

### Image pre-processing

**Fig. 1:**
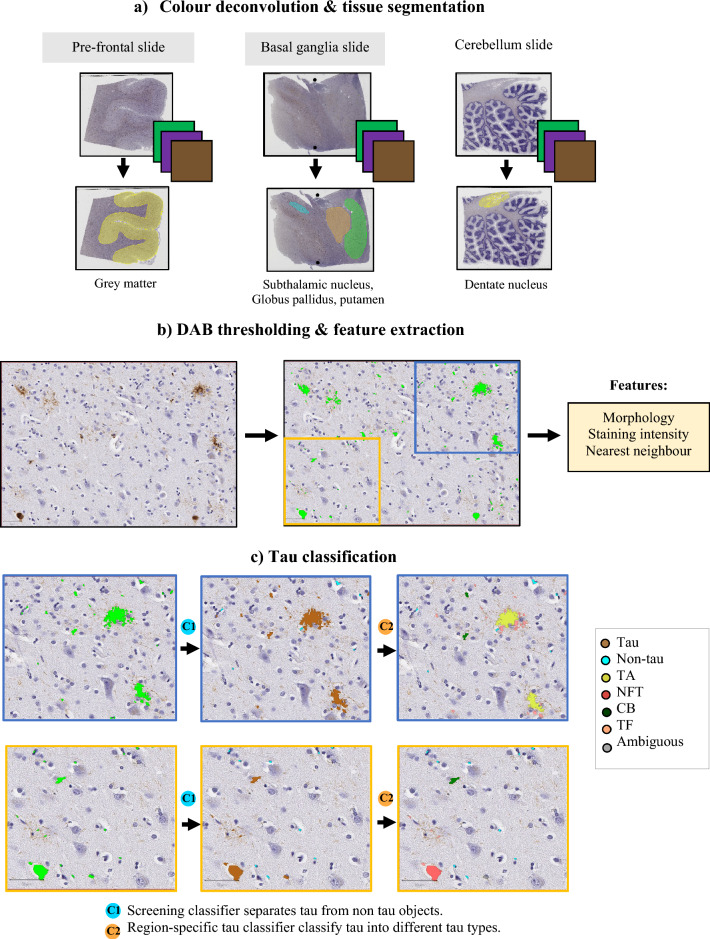
Tau pipeline overview. **a** In QuPath, a whole slide image is digitally separated into haematoxylin, DAB, and residual channels. Tissue segmentation for region of interest follows where grey matter is segmented from cortical slides, subthalamic nucleus, globus pallidus and putamen are segmented from basal ganglia slides, and dentate nucleus is segmented from cerebellum slides. Obvious artefacts are also manually removed. **b** DAB thresholding is performed to detect tau objects (in green) and features are extracted for each object. **c** Tau classification (examples presented in yellow and blue boxes for a pre-frontal slide) begins with separating non-tau artefacts from tau objects using a universal screening classifier and tau objects are then classified into different tau types using region-specific tau classifiers (which include 4 different tau classifiers, for cortical regions, putamen, subthalamic nucleus and globus pallidus, and dentate nucleus). Final slide checking is required to ensure accurate results before subsequent analysis. TA Tufted astrocyte, NFT Neurofibrillary tangle, CB coiled bodies, TF tau fragments, DAB 3,3′-diaminobenzidine

All pre-processing steps (see Fig. 1) were carried out in QuPath (version 0.4.3) software [23]. First, color deconvolution was applied to all scanned bright-field (H-DAB) whole slide images to digitally separate stains into three different channels: the DAB channel for hyperphosphorylated tau, the hematoxylin channel for cell nuclei, and a residual channel. Slides were then manually inspected to remove obvious artefacts such as DAB artefacts, de-focused regions, folded tissue, air bubbles and other confounding objects. Brain tissue was separated from the background and segmented into respective regions; for cortical regions, a semi-automated grey and white matter segmentation was carried out using the simple tissue detection tool, followed by the wand tool to manually fine-edit the segmentation. For basal ganglia regions, the putamen, globus pallidus (including internal and external part), and subthalamic nucleus were manually segmented by neuropathologists (AQ, SSK). The dentate nucleus was segmented from the cerebellum slide by a trained expert (TP).

### DAB thresholding and feature extraction

A thresholder tool in QuPath software [23] was applied to the DAB channel to detect tau objects (*resolution* = *high, pre-filter* = *Gaussian, smoothing sigma* = *0, threshold* = *0.25, minimum object size* = *5μm*^*2*^*).* Areas with DAB intensity above the threshold were labelled as tau objects. Optimal parameters of the thresholder were obtained from visual inspection to maximise the detection of tau and minimise the detection of noise and artefacts.

To reduce the creation of artefacts resulting from bleeding of digital stains between the haematoxylin and DAB channels, we applied an initial screening classifier. This is a random forest classifier trained on all extracted features to separate non-tau from tau objects. Non-tau objects include artefacts from slide preparation, and brown biological elements such as iron granules and lipofuscin.

In total, 54 features were calculated using available built-in functions in Qupath and extracted from each tau object (see Table 2). These comprised 6 morphological features and 35 intensity features, where 5 features (*minimum, maximum, mean, median and standard deviation*) were calculated from 7 channels (*red, green, blue, DAB, haematoxylin, brightness, and saturation*). Thirteen Haralick features from the DAB channel were also computed for textural information.

**Table 2:** Haralick and morphological features extracted from detected objects used in training the machine learning model

Features	
Haralick features	Angular second moment (F0)
	Contrast (F1)
	Correlation (F2)
	Sum of squares (F3)
	Inverse difference moment (F4)
	Sum average (F5)
	Sum variance (F6)
	Sum entropy (F7)
	Entropy (F8)
	Difference variance (F9)
	Difference entropy (F10)
	Information measure of correlation 1 (F11)
	Information measure of correlation 2 (F12)
Morphology	Area
	Circularity
	Length
	Maximum diameter
	Minimum diameter
	Solidity

### Training set

**Fig. 2:**
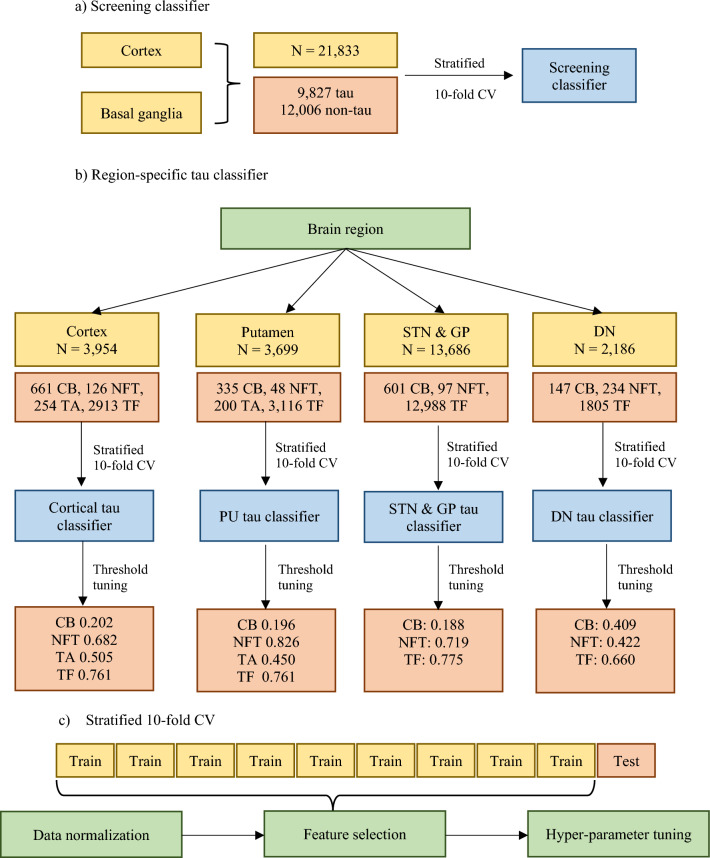
Schematic diagram showing annotated data and hyper-parameter tuning steps for the **a** screening classifier and **b** region-specific tau classifiers. **c** For each loop through the stratified tenfold cross validation (CV), data normalization, feature selection using feature recursive elimination with a random forest and the hyper-parameter tuning of the random forest were carried out

To create an equal sampling area for each training slide, a grid view was used (*grid size* = *250* × *250 mm*). Each tau object labelled by DAB thresholding was manually labelled as belonging to one of the five classes (‘coiled body’ (CB), ‘neurofibrillary tangle’ (NFT), ‘tufted astrocyte’ (TA), ‘tau fragments’ (TF), and ‘non-tau’). CB is an oligodendroglial tau inclusion with coiled-like structure and are smaller than NFT, which is a neuronal tau inclusion with elongated, flamed shape. TA is generally quite large and has a star-like tufts of densely packed fibres in astrocytes, and TF are threads or fragments of tau that were not detected as CB, NFT, or TA. A screening classifier was trained on 9827 tau and 12,006 non-tau objects annotated from cortical and basal ganglia slides (see Fig. 2).

For the cortical tau classifier, training objects were sampled from boxes defined over areas of high tau burden, yielding 3954 objects (661 CB, 126 NFT, 254 TA, 2913 TF). For basal ganglia and the dentate nucleus, 4-grid boxes with 1-grid spacing between the boxes were drawn to cover the entire area for sampling. The tau classifier for the putamen was trained on 3699 tau objects (335 CB, 48 NFT, 200 TA, 3116 TF) and the tau classifier for the subthalamic nucleus and globus pallidus was trained on 13,686 tau objects (601 CB, 97 NFT, 12,988 TF). The tau classifier for the dentate nucleus was trained on 2186 tau objects (147 CB, 234 NFT, 1805 TF). The tau classifiers for the subthalamic nucleus and globus pallidus, and dentate nucleus were not trained to detect TA as they are very rare in these regions, unlike in the putamen and cortex.

### Held-out test set

Two slides from each of the cortical, basal ganglia and dentate nucleus regions were randomly selected as held-out test slides and annotated by a trained expert (TP) and a neuropathologist (AQ). Cohen’s kappa was used to assess the inter-rater reliability alongside classification performance against the trained expert. In total, 5754 objects were annotated for cortical slides (296 CB, 78 NFT, 237 TA, 1761 TF, 3382 non-tau). For the basal ganglia, 6528 objects were annotated (153 CB, 21 NFT, 2795 TF, 44 TA, 3515 non-tau), with 2207 objects in the globus pallidus, 2199 objects in putamen, 2122 objects in the subthalamic nucleus. For dentate nucleus, 2280 objects were annotated (18 CB, 26 NFT, 844 TF, 1392 non-tau).

### Model development

Random forest algorithms are a type of tree-based ensemble algorithm that re-sample data to create many bootstrapped (smaller) datasets. A decision tree is then created for each random subset of variables for each bootstrap dataset. Random forest classification considers class prediction voting from all trees in the forest and outputs a final class prediction with the majority of votes. There are many potential extensions to the standard random forest to tackle the class imbalance issue, which can be largely grouped into two different techniques: cost-sensitive learning and re-sampling techniques [24]. The former concerns changing the weight or penalty parameters of the algorithm while the latter directly changes the class distribution by re-sampling the dataset. Re-sampling techniques have been widely shown to improve classification performance better than cost-sensitive learning techniques [18, 24]. Therefore, in this study, we used balanced random forest which randomly under-samples the majority class in each bootstrap, making the data balanced [24]. As a random forest classifier makes a final class prediction based on majority voting, it operates under the assumption that each class has equal likelihood or threshold of occurring. This can be adjusted to address severe class imbalance issue using a threshold-moving technique [25–27]. This is especially relevant for tau burden classification as their relative proportions are different in cortical and subcortical structures [2].

### Hyper-parameter tuning

The Sci-kit learn (version 0.24.1) [28] and Imbalanced-learn (version 0.8.1) [29] libraries in Python (version 3.9.7) were used to implement a random forest algorithm for the tau classification pipeline. The data was standardised (*mean* = *0, SD* = *1*) and tenfold stratified cross validation was used to train the classifiers, partitioning data into 10 folds (see Fig. 2). At each iteration, 9 out of 10 folds were used as training data and one-fold was used to validate training performance.

In the balanced random forest classifier, each bootstrap sample was class balanced. During the training phase, feature selection was performed using recursive feature elimination. Hyper-parameters of the balanced random forest were tuned using a random-search with the following parameter space: *n_features_to_select* = *[28, 30, 34, 36, 38, 40, 42, 44, 46, 48, 50, 52, 54]* (number of features to select)*, n_estimators* = *[100, 200, 300, 400, 500, 600, 700, 800, 900, 1000]* (number of trees in the forest), *max_features* = *[0.2, 0.4, 0.6, 0.8, 1]* (number of features to consider for best split), *max_depth* = *[5, 10, 15, 20, None]* (maximum depth of the tree), *min_samples_split = [2, 5, 10]* (minimum samples required to split further), *min_samples_leaf = [1, 2, 4]* (minimum samples required to be a leaf node), *sampling_strategy* = *[‘auto’, ‘all’, ‘not majority’, ‘majority’]* (sampling strategy to sample the dataset), *max_samples* = *[0.25, 0.5, 0.75, None]* (number of bootstrap samples to draw to train each base estimator), *class_weight* = *[‘balanced’]* (weight or importance associated with the classes). The balanced random forest was optimised based on the mean area under the 4 precision-recall curves (PR-AUC) using a one-vs-rest approach (TA vs. rest, CB vs. rest, NFT vs. rest, TF vs. rest).

### Class-specific threshold tuning

Using the hyper-parameters found, optimal class-specific thresholds were tuned to tackle class imbalance. Predicted class scores were used to re-compute PR-AUC using a one-vs-rest approach as above. The PR-AUC for each class was optimised using the F1-score. After obtaining class-specific thresholds, class probabilities for each object were thresholded to obtain the predicted class label. Brain regions with similar tau morphology and distribution were grouped together where class-specific thresholds were tuned separately for each regional grouping. In this study, there were 4 regional groupings: cortex, putamen, globus pallidus and subthalamic nucleus, and dentate nucleus.

If an object’s class probability passed the class-specific threshold, an object would be labelled as the corresponding class. To mirror human classification of tau objects, we assessed the ambiguity of tau object classification. If more than one class or no class passed the class-specific threshold, the object was labelled as ‘Ambiguous’ and discarded from further analyses.

After classification, the precision, recall, macro F1-score and confusion matrix of the model were collected. The model was then applied to the held-out test set to evaluate its performance generalisability. Finally, the optimised model was applied to the remaining novel slides to perform tau classification and quantification for further analyses.

### Tau quantification

The four types of tau quantified were CB, NFT, TA and TF. This enabled the calculation of total tau and tau hallmarks (all tau excluding TF). Using raw counts of tau quantified, tau density was calculated as the number of tau objects per unit area (μm^2^) of the region quantified. For cortical regions, tau density was quantified in cortical grey matter, while the entire nuclei area was used for basal ganglia and dentate nucleus.

### Correlation with PSP staging

Polar plots using the *plotly* package in Python [30] were used to show regional tau distribution quantified from the pipeline for both total tau and tau density by tau type. Spearman's rank correlation coefficient was used to compute the correlation between tau density quantified across regions and PSP stage. Correlations between region-specific tau density and region-specific rating were also computed within regions of the PSP staging scheme.

### Clinicopathological correlations

For this analysis, we included 28 PSP subjects with available PSPRS scores and due to the skewness of tau density distribution, a logarithmic transformation (log_10_) was applied to tau density. To investigate the relationship between *postmortem* tau and PSPRS score, the *brms* package in R (version 1.4.1717) [31–33] was used to construct Bayesian linear mixed regression models. Bayesian analysis enables the calculation of posterior probability distributions showing the uncertainty of the regression coefficient estimates based on effect size [34], and permits the null hypothesis to be rejected or accepted [34]. The analysis was first carried out with PSP stage as the predictor, PSPRS total score as the outcome variable, and disease duration and PSPRS to death interval as covariates to establish a baseline relationship between the staging scheme and PSPRS score. The same analysis was repeated with tau density quantified from all regions and separately from only cortical and subcortical regions as the predictor. To test whether tau type-specific burden was more informative of PSPRS score than total tau burden, total tau and tau type-specific models were created for model comparison. To estimate the strength of evidence in favor of the tau type-specific models against the total tau model, we used a standard Bayes Factor (BF) cut-off of 3 to indicate at least moderate evidence [35]. In the final model, the strength of regression coefficient was assessed using the Region of Practical Equivalence (ROPE). Given the optimal ROPE is not established a priori, we used a standard approach to define the ROPE as a range of values ± 0.1 of the standard deviation of a standardized parameter (PSPRS score) [36]. If 95% of the credible interval (Crl) of the regression coefficient falls completely within the ROPE, then the effect of the parameter would be equivalent to the null value for practical purposes [35, 37].

A Gaussian model family was selected based on the distribution of the data. A weakly informative normal prior *(mean* = *0, SD* = *100)* was chosen for the regression coefficients and default priors were used for the intercept *(student-t prior; df* = *3, mean* = *53.5, SD* = *12.6)* and the sigma *(student-t prior; df* = *3, mean* = *0, scale* = *12.6).* The model configuration was the same for all models (warmup = 10,000, iteration = 20,000). All models went through prior and posterior predictive checks to ensure that the configurations were appropriate. All models converged with no divergences or diagnostic warnings, and in all cases R^^^ convergence values were ~ 1.00 (see Additional file 1). Due to the complexity of our analysis, sensitivity analysis of priors was conducted to only assess the effect of prior choice on neuropathological severity (PSP stage, tau burden) in the final models. We chose two other weakly informative normal priors, one more informative *(mean* = *0, SD* = *50)* and the other less informative *(mean* = *0, SD* = *150)* to assess the sensitivity of posterior estimates on the prior choice.

## Results

### Classification performance

#### Hyper-parameter tuning

**Fig. 3:**
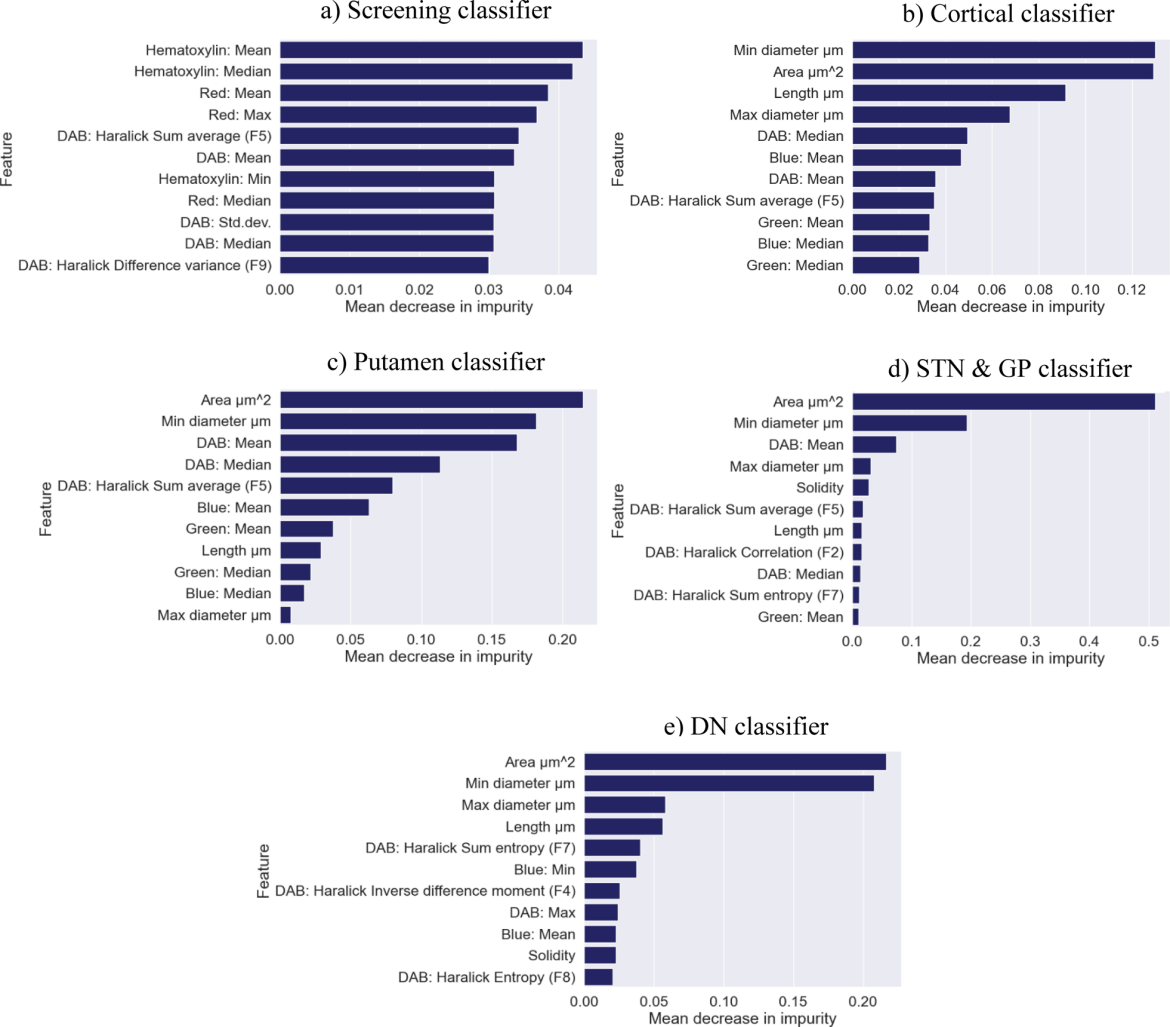
Top ten most important features based on mean decrease in impurity of each classifier from hyper-parameter tuning. **a** screening classifier, **b** cortical tau classifier, **c** tau classifier for putamen, **d** tau classifier for subthalamic nucleus and globus pallidus (STN & GP), and **e** tau classifier for dentate nucleus (DN)

All classifiers were optimised for PR-AUC where tau classifiers for different regions yielded different set of optimal hyperparameter values, except for the classifiers for basal ganglia nuclei (see Table 3). All classifiers achieved PR-AUC scores of over 0.97 (Table 4) where the screening classifier achieved the highest PR-AUC of 0.99, and the tau classifier for the subthalamic nucleus and globus pallidus achieved the lowest PR-AUC of 0.97. Tau classifiers for the cortex, basal ganglia nuclei and dentate nucleus achieved similar PR-AUC scores of 0.98. Tau classifiers for non-cortical regions selected 34 from 54 features, while 40 features were selected for the cortical tau classifier and 46 features were selected for the screening classifier from the hyper-parameter tuning step. For feature importance (Fig. 3), the top ten most important features for the screening classifier were mainly staining intensities in hematoxylin, red and DAB channels. Tau classifiers for different brain regions showed the same trend where morphological features such as area and diameter of tau objects were the most important, followed by staining intensities and textural features.

**Table 3:** Selected parameter from hyper-parameter tuning using stratified tenfold cross validation for the screening and region-specific tau classifiers

Parameter	Screening	Cortical	Putamen	STN & GP	Dentate nucleus
N_features_to_select	46	40	34	34	34
Sampling strategy	‘auto’	‘not majority’	‘not majority’	‘not majority’	‘not majority’
n_estimator	600	800	500	500	100
min_sample_split	2	2	2	2	2
min_sample_leaf	2	1	2	2	1
max_features	1	0.2	0.6	0.6	0.2
max_depth	None	10	15	15	None
max_sample	None	0.75	0.75	0.75	None

Hyperparameters determine machine learning model architecture and are chosen before training. Hyperparameter tuning, which is part of training, is carried out to search for an optimal set of model parameters. *STN* Subthalamic nucleus; *GP* Globus pallidus

**Table 4:** Classification performance with and without threshold-moving method from training for screening and subsequent tau classifiers

Classifier	Precision	Recall	F1-score	PR-AUC
*Without threshold-moving approach*				
Screening	0.96 (± 0.044)	0.96 (± 0.053)	0.96 (± 0.060)	0.99 (± 0.011)
Cortex	0.92 (± 0.037)	0.92 (± 0.017)	0.91 (± 0.027)	0.98 (± 0.010)
PU	0.90 (± 0.046)	0.86 (± 0.068)	0.86 (± 0.061)	0.98 (± 0.015)
STN & GP	0.93 (± 0.038)	0.86 (± 0.056)	0.87 (± 0.061)	0.97 (± 0.020)
DN	0.96 (± 0.020)	0.93 (± 0.041)	0.94 (± 0.029)	0.98 (± 0.016)
*With threshold-moving approach*				
Screening	0.96 (± 0.046)	0.96 (± 0.057)	0.95 (± 0.064)	0.99 (± 0.011)
Cortex	0.95 (± 0.025)	0.95 (± 0.027)	0.95 (± 0.021)	0.98 (± 0.010)
Putamen	0.94 (± 0.024)	0.92 (± 0.049)	0.93(± 0.037)	0.98 (± 0.015)
STN & GP	0.95 (± 0.026)	0.92 (± 0.042)	0.93 (± 0.027)	0.97 (± 0.020)
DN	0.96 (± 0.028)	0.95 (± 0.026)	0.95 (± 0.023)	0.98 (± 0.016)

Tau classifiers for the cortex, putamen (PU), subthalamic nucleus and globus pallidus (STN & GP), and dentate nucleus (DN). Classifiers were tuned for area under the precision-recall curve (PR-AUC), where precision, recall and F1-score were calculated. Mean values from cross-validation and standard deviation in brackets are reported

#### Threshold-moving optimisation

After optimising the hyper-parameters for each classifier, the next step was to tune class-specific thresholds for assigning labels to individual tau objects. Using a one-vs-rest approach, the class threshold with the highest F1-score was selected (Table 5). The threshold for tau (threshold = 0.46; F1-score 0.97) was lower than non-tau (threshold = 0.53; F1-score 0.97) in the screening classifier. The threshold for TF was the highest for the cortical tau classifier, followed by NFT, TA and CB classes. The class thresholds of the tau classifier for the putamen followed a similar pattern but differed in that the NFT threshold was higher than the TF class. The tau classifier for the subthalamic nucleus and globus pallidus and dentate nucleus followed the same trend, where the TF class threshold was highest, followed by the NFT and CB classes.

**Table 5:** Class-specific thresholds of tau classifiers

Classifier	CB	F1-score	NFT	F1-score	TA	F1-score	TF	F1-score
Cortex	0.20	0.95	0.68	0.91	0.51	0.96	0.76	0.99
Putamen	0.20	0.88	0.83	0.98	0.45	0.94	0.76	0.99
STN/GP	0.19	0.90	0.72	0.95	N/A	N/A	0.78	1.00
DN	0.41	0.91	0.42	0.98	N/A	N/A	0.66	0.99

Tau classifiers for the cortex, putamen, subthalamic nucleus and globus pallidus (STN & GP) and dentate nucleus (DN). Thresholds were optimised for F1-score using a one-vs-rest approach. Not applicable (N/A) is reported where TA is not quantifiable

We further compared the classification performance of the threshold-moving method to the default method of assigning class labels based on maximum class scores. The screening classifier with or without the threshold-moving method performed similarly, with F1-scores of 0.96 and 0.95 respectively. Therefore, the screening classifier without threshold-moving was selected as the final screening model. For tau classifiers, the threshold-moving method improved the mean F1-score and were used in the final models (Table 4).

#### Final models and confusion matrices

**Fig. 4:**
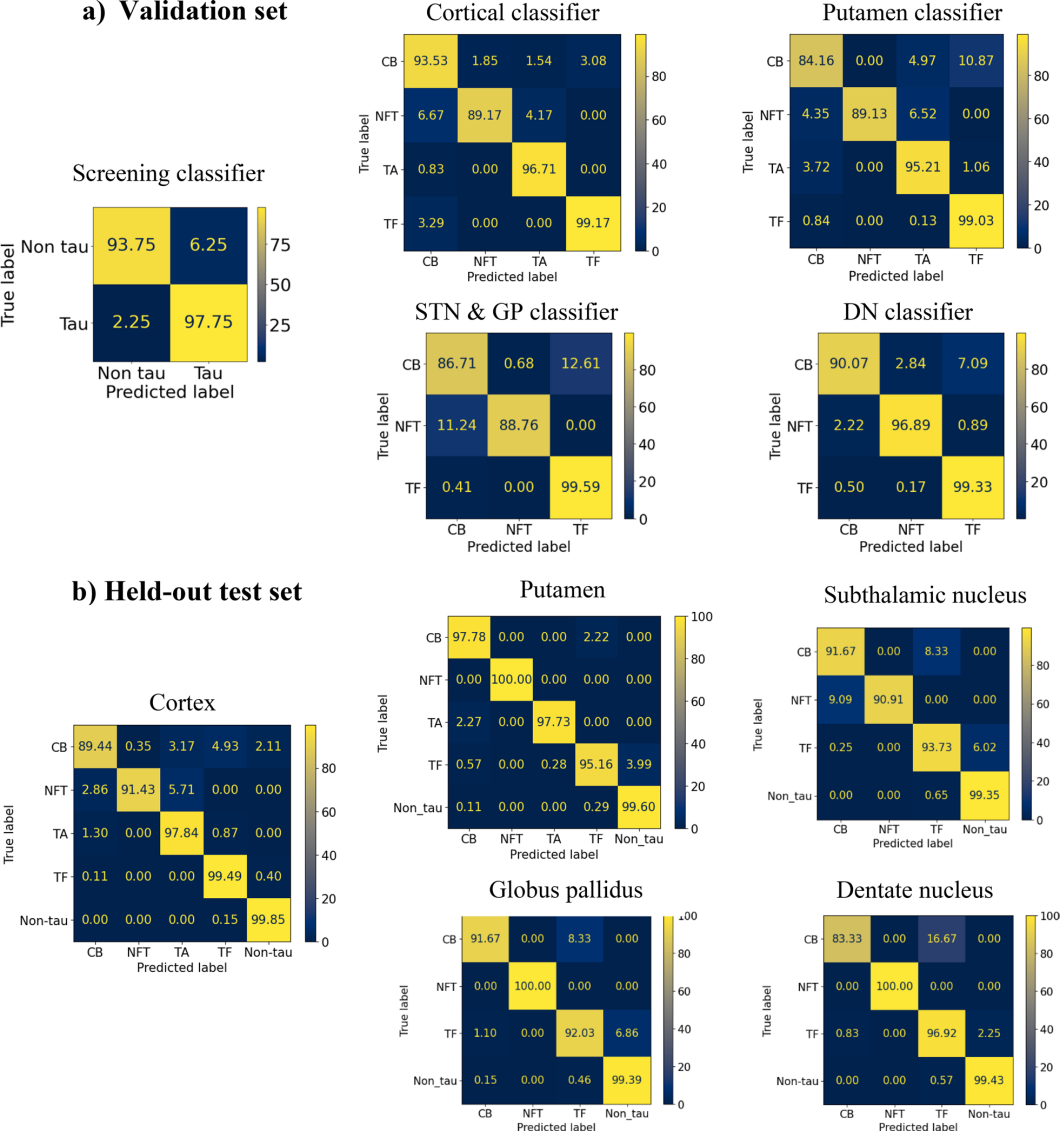
Mean confusion matrices are presented **a** for each classifier from the validation set in the tenfold cross validation, and **b** for each brain region in the **b** held-out test set

Confusion matrices for each classifier from validation set are shown in Fig. 4**.** The screening classifier achieved high accuracy for both tau (97.75%) and non-tau (93.75%) with minimal misclassification. Tau classification for cortical regions achieved the highest accuracy of 99.17% in classifying TF correctly, followed by TA (96.71%), CB (93.53%) and NFT (89.17%). NFT was misclassified as CB most often (6.67%) while CB was most often misclassified as TF (3.08%). Similarly, tau classification for the putamen achieved the highest accuracy in classifying TF (99.03%) followed by TA (95.21%). However, the classifier misclassified CB most often (accuracy 84.16%) as opposed to NFT (accuracy 89.13%). CB was most wrongly classified as TF (10.87%) but not vice versa. NFT was wrongly classified as either TA (6.52%) or CB (4.35%), but never as TF whilst TA was most often classified as CB (3.72%). For tau classifiers in regions with no TA quantified, they performed best in classifying TF correctly (99.33% for dentate nucleus, 99.59% for subthalamic nucleus and globus pallidus). Tau classification was slightly lower in the subthalamic nucleus and globus pallidus in classifying NFT (88.76% vs. 96.89%) and CB (86.71% vs. 90.07%) correctly, compared to the dentate nucleus. For misclassifications, a similar pattern was seen in both regions where CB was mostly misclassified as TF and NFT, while TF were rarely misclassified. The proportion of objects labelled as ‘Ambiguous’ from each of the tau classifiers was no more than 1% of tau objects.

#### Validation on the held-out test set

**Fig. 5:**
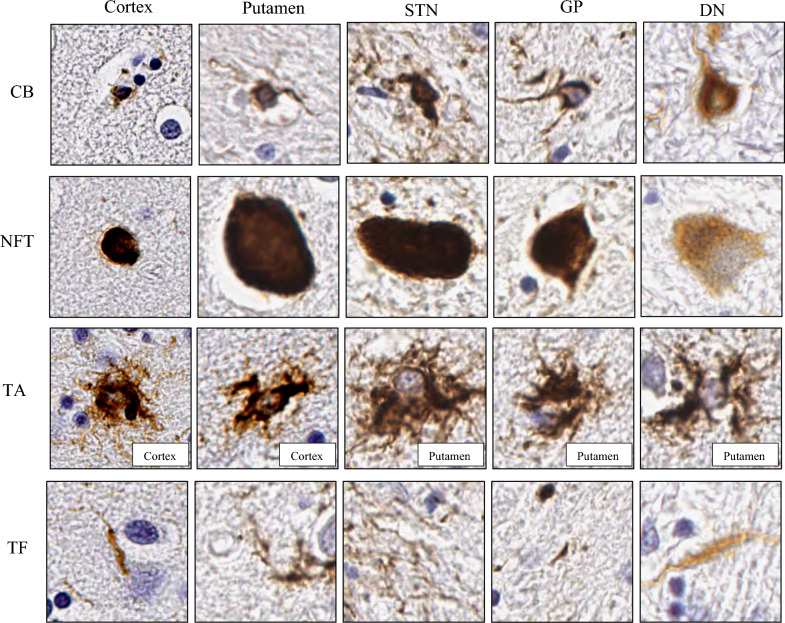
Examples of correct classification from the held-out test set for each tau aggregate type from cortical, putamen, subthalamic nucleus (STN), globus pallidus (GP) and dentate nucleus (DN). All images were cropped 150 × 150 mm window size. TA examples are only drawn from cortical and putamen. *CB* coiled body, *NFT* neurofibrillary tangle, *TA* tufted astrocyte, *TF* tau fragments

From Table 6, using rater 1 as the ground truth (the trained expert), the F1-score of the classification performance in each of the brain regions ranged from 0.92 to 0.98. The classifier performed best in the putamen, followed by the cortex, globus pallidus, dentate nucleus and subthalamic nucleus. Furthermore, Cohen’s kappa indicated that the agreement between rater 1, the algorithm and rater 2 across brain regions was high, at least 0.87. The agreement between the two human raters was higher than the algorithm and rater 1 across all regions, where the smallest difference was by 0.02 in the cortex and putamen, followed by 0.03 difference in dentate nucleus, 0.06 in globus pallidus and 0.13 difference in the subthalamic nucleus, indicating levels of classification uncertainty in each region. Looking at the confusion matrices for each region in the held-out test set (Fig. 4), the algorithm achieved above 90% accuracy in classifying tau types across all brain regions but struggled more with classifying CB accurately in the cortex (89.44%) and dentate nucleus (83.33%) as they could be mistaken for TF. Figure 5 displays examples of correct classification of tau type-specific aggregates across all brain regions. CB has a coiled-like structure and can appear larger in subcortical structures compared to the cortex. Similarly, NFT is a highly pigmented oval structure and can appear larger in subcortical structures, particularly the subthalamic nucleus, than in the cortex. The dentate nucleus has numerous pre-tangles which are generally more diffuse and granular than NFT and may be detected as NFT in the pipeline. Correctly classified TA have star-like tufts of densely packed fibres and appear larger than CB and NFT in general. TF consists of threads and background tau burden that can often be difficult to associate with a cell.

**Table 6:** Classification performance on a held-out test set

Region	Precision	Recall	F1-score	Algorithm & rater 1	Rater 1 & 2
Cortex	0.98	0.96	0.97	0.94	0.96
Putamen	0.97	0.98	0.98	0.97	0.99
Subthalamic nucleus	0.91	0.94	0.92	0.87	1.00
Globus pallidus	0.95	0.96	0.96	0.93	0.99
Dentate nucleus	0.91	0.95	0.93	0.97	1.00

Precision, recall and F1-score are reported and supplemented with Cohen’s kappa to show agreement between the raters (algorithm, rater 1, rater 2)

### Comparison with manual semi-quantitative PSP pathology staging

#### Tau density across cortical and subcortical regions

**Fig. 6:**
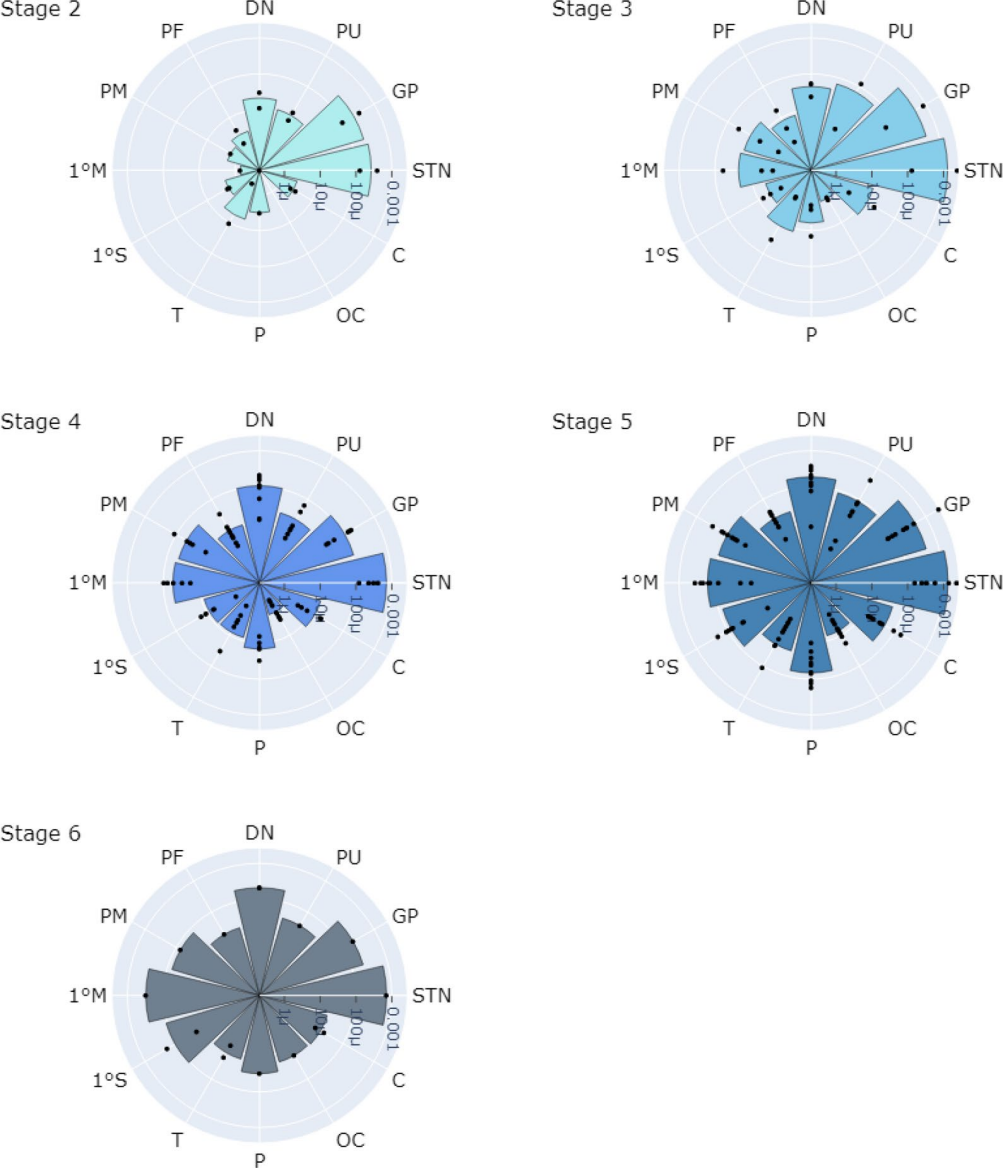
Logarithmic total tau density plot from PSP stage 2 (least severe) to 6 (most severe) across all PSP subjects from both cortical and subcortical structures. *STN* subthalamic nucleus, *GP* globus pallidus, *PU* putamen, *DN* dentate nucleus, *PF* pre-frontal, *PM* pre-motor, *1 M* primary motor, *1 S* primary somatosensory, *T* temporal, *P* parietal, *OC* occipital, *C* cingulate

Tau was quantified and cases were grouped based on their PSP pathology stage, shown in Fig. 6. Tau pathology density in subcortical regions was greater than in cortical regions, in keeping with the tau staging system suggesting earlier subcortical involvement. In stage 2, tau accumulation was most prevalent in the subthalamic nucleus and globus pallidus, followed by the dentate nucleus and putamen with minimal tau in cortical regions. In stage 3, there was greater tau pathology in subcortical regions and tau could be seen across multiple cortical regions, especially in the frontal regions, while tau pathology in the occipital lobe was minimal. From stages 4 to 6 tau pathology was greatest in subcortical regions, particularly the subthalamic nucleus and globus pallidus, but the density of tau pathology in the cortical areas increased with each stage, particularly in the frontal lobe.

**Fig. 7:**
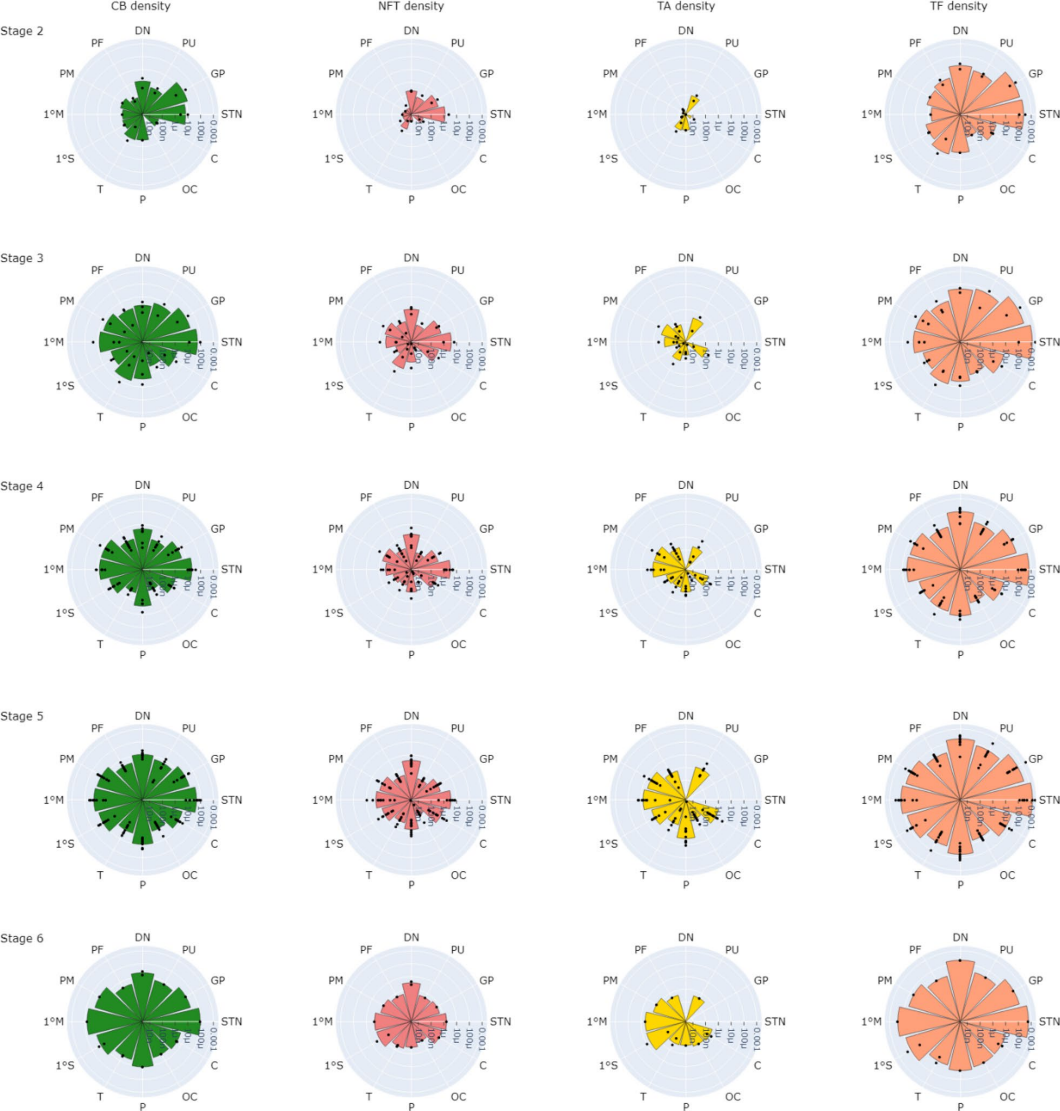
Logarithmic tau density plot by tau type per PSP stage from stage 2 (top) to 6 (bottom) across all PSP subjects and brain regions sampled. CB density plot (green), NFT density plot (red), TA density plot (yellow) and TF density plot (orange). *STN* subthalamic nucleus, *GP* globus pallidus, *PU* putamen, *DN* dentate nucleus, *PF* pre-frontal, *PM* pre-motor, *1 M* primary motor, *1 S* primary somatosensory, *T* temporal, *P* parietal, *OC* occipital, *C* cingulate, *CB* coiled body, *NFT* neurofibrillary tangle, *TA* tufted astrocyte, *TF* tau fragments

In the cortex, frontal regions including primary motor and pre-motor regions were most severely affected. Temporal and parietal regions showed tau accumulation but to a lesser degree than frontal regions, while the occipital region still showed the least accumulation of tau. Examining tau type-specific density plots (Fig. 7), the density of tau fragments was higher than other tau types across all PSP stages.

When focusing on individual tau hallmarks (not including TF), CB density was the most abundant tau type, followed by NFT density, then TA density, across PSP stages. In stage 2, CB and NFT densities followed the general pattern of total tau accumulation where they were predominantly found in subcortical regions, specifically the globus pallidus and subthalamic nucleus. From stage 3–6, CB and NFT appeared in cortical regions and continued to accumulate in subcortical regions. The main cortical regions with high CB and NFT densities included motor and parietal regions, with the least affected area being the occipital region.

TA density was highest in the putamen and could be observed in cortical regions. In contrast to CB and NFT, TA density was the highest in the putamen in stage 2 and was minimal in cortical regions. As the stage progressed, TA density increased predominantly in the putamen and cortical regions, where TA density in cortical regions showed the same pattern as NFT and CB accumulation.

#### Correlation to the current PSP staging scheme

Across all brain regions in the study, there was a positive correlation between tau hallmark (CB + NFT + TA) density quantified from all regions and the overall PSP stage (Table 7). CB and TA densities showed the strongest correlation to PSP stage when considering only cortical structures. NFT density also generally showed positive correlation to PSP stage, but the correlations were weaker than that of the glial tau.

**Table 7:** Spearman’s correlation coefficients between tau density and PSP stage

Tau type	All regions	Cortical regions	Subcortical regions
Total tau density	0.37**	0.57**	0.27*
CB + NFT + TA density	0.47**	0.59**	0.39**
CB density	0.46**	0.58**	0.38**
NFT density	0.37**	0.51**	0.24*
TA density	0.37**	0.62**	N/A
TF density	0.36**	0.56**	0.26*

Correlation coefficients are reported when considering all brain regions, only cortical regions, and only subcortical regions. *Correlations significant at *P* < 0.05, **Correlations are significant at *P* < 0.001. Not applicable (N/A) as TA density is only quantifiable in putamen. *CB* coiled body*, NFT* neurofibrillary tangle*, TA* tufted astrocyte*, TF* tau fragments

Next, we investigated the contribution of tau quantified at each region in the PSP staging system (Table 8) to the overall PSP stage. Total tau and tau hallmark density in the occipital region showed the highest correlation to the overall PSP stage, followed by pre-frontal, dentate nucleus, subthalamic nucleus, putamen and globus pallidus respectively. These trends are in-line with the defining features of PSP staging where subcortical regions are heavily affected early in the disease stage therefore tau density in these regions is less informative in distinguishing between higher PSP stages than tau density in cortical regions that is a feature of mid to late disease stages.

**Table 8:** Spearman’s correlation coefficients between tau density and the overall PSP stage

Tau type	GP	STN	PU	DN	PF	OC
Total tau density	0.19 (*p* = 0.41)	0.34 (*p* = 0.13)	0.28 (*p* = 0.22)	0.63**	0.70**	0.85**
Tau hallmark density	0.30 (*p* = 0.19)	0.51*	0.37 (*p* = 0.10)	0.71**	0.75**	0.81**
CB density	0.30 (*p* = 0.19)	0.49*	0.37 (*p* = 0.10)	0.72**	0.67**	0.79**
NFT density	0.12 (*p* = 0.61)	0.12 (*p* = 0.60)	0.53*	0.68**	0.54*	0.69**
TA density	N/A	N/A	0.33 (*p* = 0.14)	N/A	0.73**	0.83**
TF density	0.16 (*p* = 0.48)	0.34 (*p* = 0.13)	0.27 (*p* = 0.24)	0.60*	0.67**	0.84**

Tau density was quantified from each region in the PSP staging system. *GP* Globus pallidus, *STN* subthalamic nucleus, *PU* putamen, *DN* dentate nucleus, *PF* pre-frontal and *OC* occipital region. *Correlations are significant at *p* < 0.05, **Correlations are significant at *p* < 0.001. Not applicable (N/A) where TA density is not quantifiable. *CB* coiled body*, NFT* neurofibrillary tangle*, TA* tufted astrocyte*, TF* tau fragments

When looking at individual tau type-specific densities, CB density in the globus pallidus/subthalamic nucleus and dentate nucleus showed the strongest contribution in comparison to other tau types to PSP stage. In contrast, NFT density in putamen, TF and TA density in the occipital region, and TA density in the pre-frontal region showed the strongest contribution to overall PSP stage when compared to other region-specific tau densities.

Finally, we investigated the correlation between the region-specific tau density and the manually assessed region-specific severity rating to understand which tau type is most contributory to grading the severity of each region (Table 9). TF density in the basal ganglia nuclei showed the strongest positive correlation to region-specific severity rating when compared to other tau types. CB density in the dentate nucleus and TA density in cortical regions showed the highest correlation to manually rated region-specific severity. The correlation between NFT density and region-specific rating was lower than that of glial density across all regions. In general, total tau and tau hallmark density showed similar correlation strength to region-specific severity rating as the tau type-specific density that has the highest correlation to region-specific density.

**Table 9:** Spearman’s correlation coefficients between tau density and region-specific severity rating

Tau type	GP	STN	PU	DN	PF	OC
Total tau density	0.69*	0.66*	0.87**	0.51*	0.83**	0.84**
Tau hallmark density	0.62*	0.44 (*p* = 0.06)	0.83**	0.58*	0.73**	0.80**
CB density	0.62*	0.50*	0.79**	0.63*	0.63*	0.79**
NFT density	0.50*	0.20 (*p* = 0.42)	0.61*	0.56*	0.44*	0.72**
TA density	N/A	N/A	0.87**	N/A	0.81**	0.84**
TF density	0.67*	0.66*	0.87**	0.48*	0.81**	0.82**

Measures were quantified from each region in the PSP staging system. *GP* Globus pallidus, *STN* subthalamic nucleus, *PU* putamen, *DN* dentate nucleus, *PF* pre-frontal and *OC* occipital region. *Correlations are significant at *p* < 0.05, **Correlations are significant at *p* < 0.00. Not applicable (N/A) where TA density is not quantifiable. *CB* coiled body*, NFT* neurofibrillary tangle*, TA* tufted astrocyte*, TF* tau fragments

#### PSP stage, tau burden and PSPRS scores

**Fig. 8:**
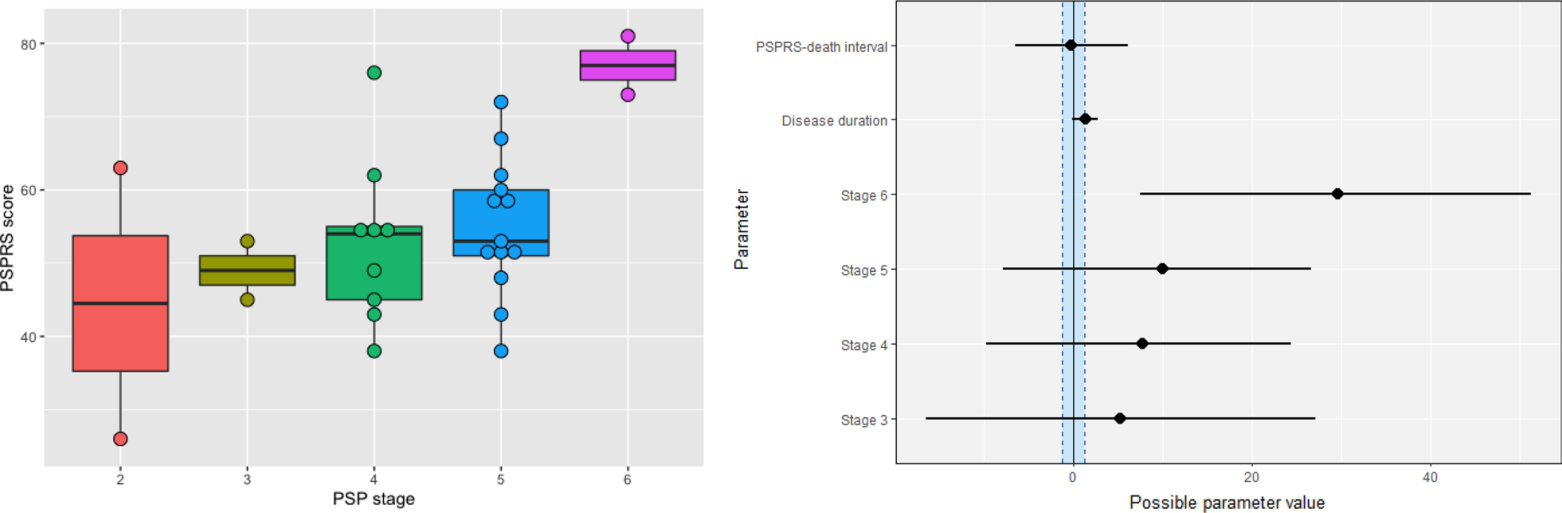
A boxplot showing PSPRS score and PSP stage (left) and a plot showing posterior distribution of the regression coefficients of the model PSPRS score ~ PSP stage + disease duration + PSPRS-death interval (right). Median (circle) and 95% credible interval (line) are plotted for each parameter alongside ROPE [− 1.24 to 1.24] (blue region)

Firstly, we assessed whether there was a relationship between clinical severity (using the last PSPRS score prior to death) and neuropathological severity (using the PSP pathology stage at *postmortem*). Looking across PSP stages (Fig. 8), there was evidence that the PSPRS score of stage 6 patients was higher than stage 2 patients *(median* = *28.44, Crl 6.71 to 48.57)*, while there was insufficient evidence that the PSPRS score differed between stage 3–5 patients *versus* stage 2 patients.

**Fig. 9:**
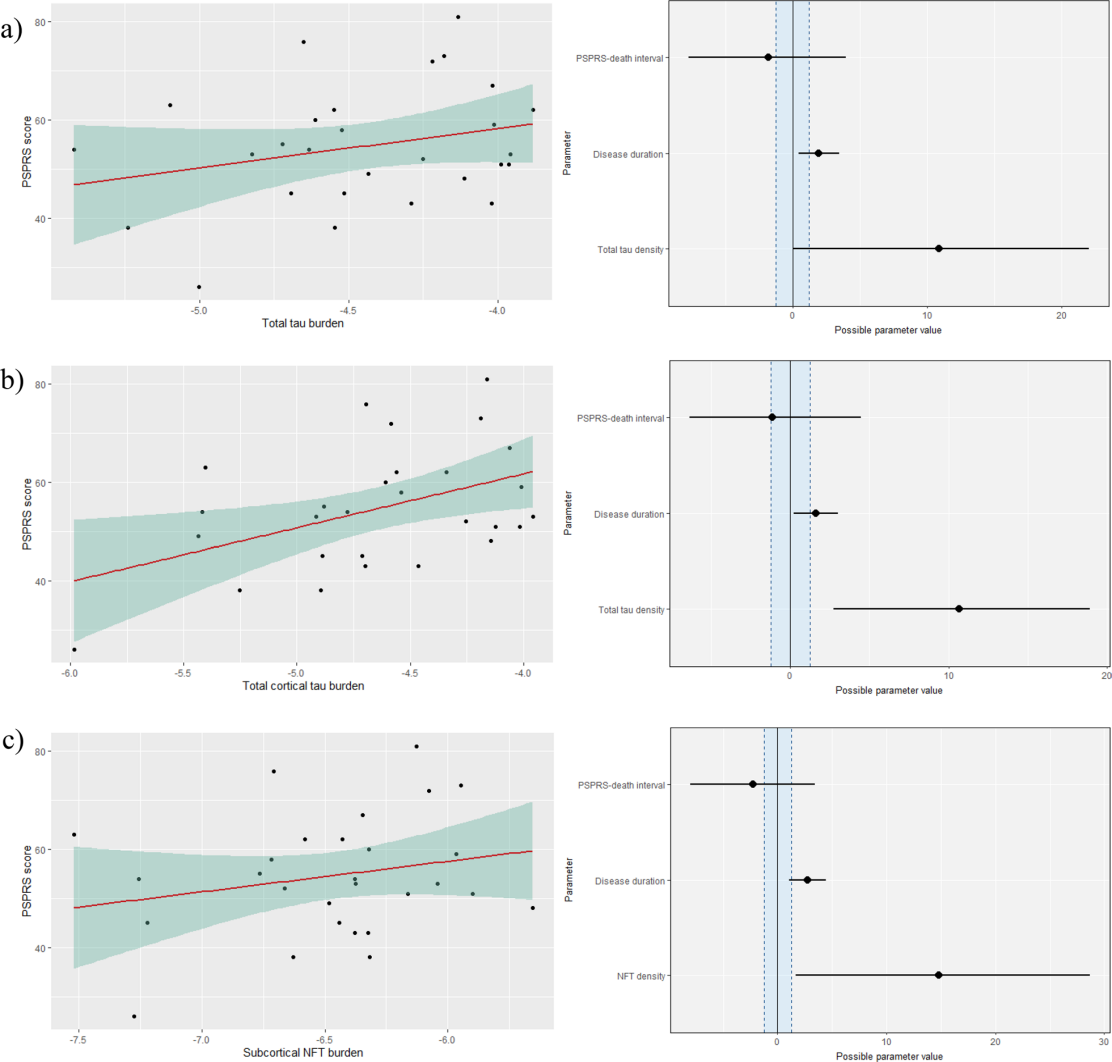
**a** A scatterplot showing PSPRS score, and total tau density quantified from all regions (left), and a plot showing the posterior distribution of the regression coefficients of the final model PSPRS score ~ total tau density + disease duration + PSPRS-death interval with possible parameter values (right). Mean (circle) and 95% credible interval are plotted with ROPE [− 1.24 to 1.24] (blue region). Plots from the final model when tau was quantified from only cortical regions with ROPE [− 1.24 to 1.24] (**b**) and only subcortical regions with ROPE [− 1.26 to 1.26] (**c**) are also presented

Next, tau type-specific models were compared against a total tau model to assess whether tau type-specific burden is more informative about the PSPRS score than the total tau burden (Table 10). Total tau burden and tau type-specific burden were equally predictive of the PSPRS score when tau was quantified from all regions or only cortical regions. However, when tau was quantified from only subcortical regions, NFT density was a better predictor of the PSPRS score than total tau burden (BF = 10.52). Upon final model inspection (Fig. 9), there was decisive evidence supporting a higher tau burden and PSPRS score when tau was quantified from either only cortical or subcortical regions. Total tau burden quantified from cortical regions only (median = 10.68, Crl 2.66 to 18.91) and NFT burden quantified from subcortical regions only (median = 14.81, Crl 1.89 to 28.50) were positively associated with the PSPRS score. Despite a positive trend between total tau burden quantified from all regions, there was insufficient evidence to support its relationship with the PSPRS score (median = 10.96, Crl − 0.24 to 21.65, 1.87% in ROPE).

**Table 10:** Bayes’ factor for the comparison between type-specific tau and total tau model in predicting PSPRS

Model	All regions	Cortical regions	Subcortical regions
CB density	0.69	0.29**	0.47
NFT density	0.82	0.29**	10.52**
TA density	0.84	0.31**	N/A
TF density	0.83	1.10	0.95

The comparison is made in 3 regional groupings; logarithmic tau density quantified across all brain regions, and separately for cortical and subcortical regions. **Indicates BF > 3 (substantial evidence for tau type-specific density that it correlates better with PSPRS score than total tau density) or BF < 1/3 (substantial evidence for total tau model as compared to tau type-specific model). Where 1/3 < BF < 3, the evidence from the available data is inconclusive. Not applicable (N/A) as TA density is only quantifiable in putamen. *PSPRS* PSP rating scale. *BF* Bayes’ factor, *CB* coiled body*, NFT* neurofibrillary tangle*, TA* tufted astrocyte*, TF* tau fragments

#### Sensitivity analysis of prior

**Fig. 10:**
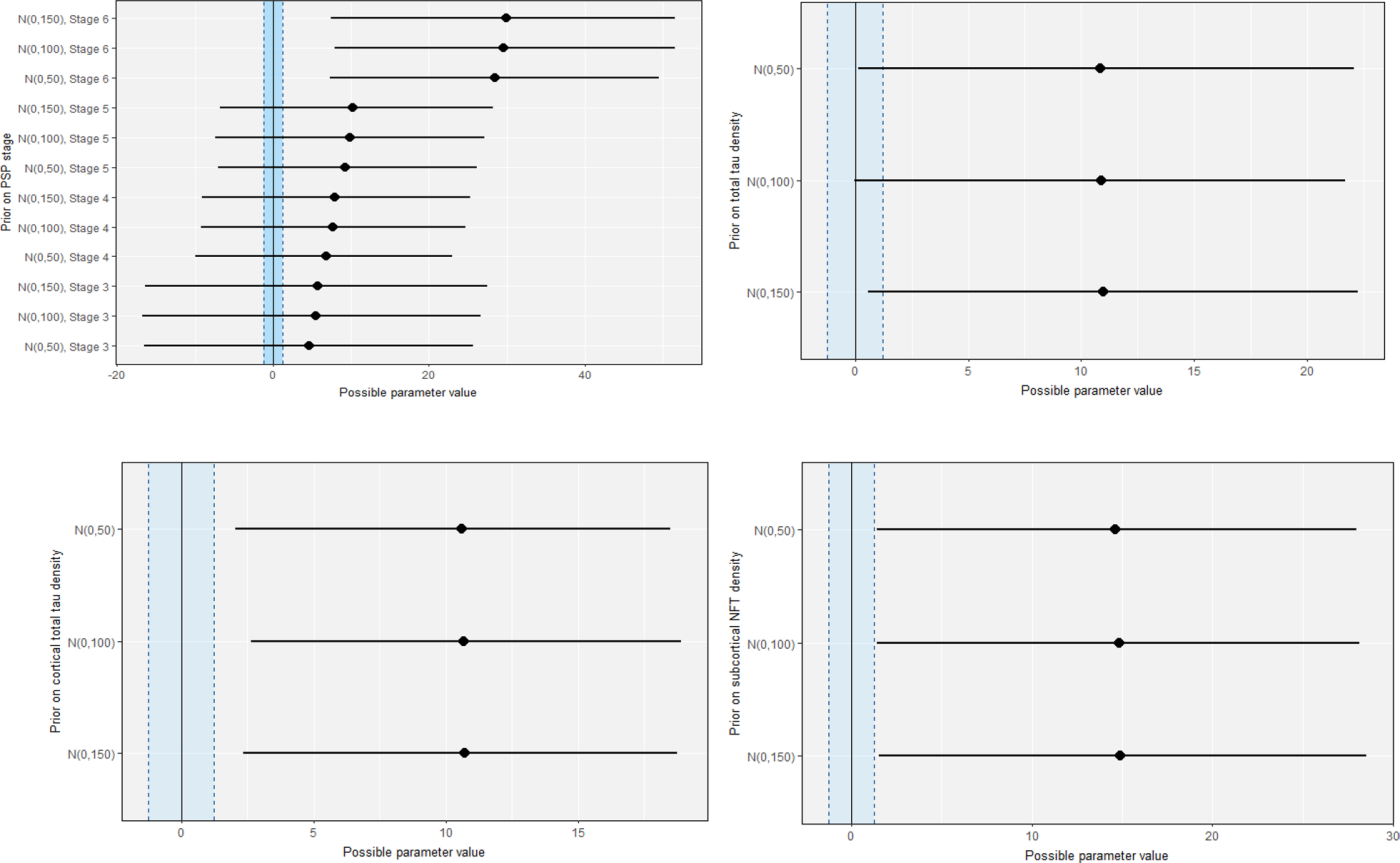
Sensitivity analysis plots showing the effect of setting alternative weakly informative priors on the regression coefficient of the effect of interest (PSP stage, tau burden) in the final models are presented. Normal distribution [N (mean, standard deviation)] was chosen with mean centred at zero, and standard deviation of  50 (more informative), 100 (the chosen value) and 150 (less informative)

We assessed the sensitivity of the posterior distribution of the effects of interest (neuropathological severity) from the chosen prior choice of N (0, 100) by setting other weakly informative priors. Due to the complexity of our analysis, sensitivity analysis was only conducted on the final models and posterior distributions of the neuropathological severity was qualitatively assessed. Figure 10 shows that choosing a less broad prior of N (0, 50) or a more broad prior of N (0, 150) does not substantially change the conclusion of the analysis when considering ROPE: the results are robust across other weakly informative prior choices.

## Discussion

We have developed a robust and reliable digital pipeline for quantification of *postmortem* tau pathology in PSP, achieving an accuracy comparable to expert assessment. The main strengths of our pipeline are its versatility permitting accurate assessment in multiple brain areas, and scalability allowing assessment across a large number, wide range of brain regions and high number of subjects. The accuracy of the method was similar for neuronal and glial tau pathology densities. We go beyond former proof of concept studies, which generally include a small subset of brain regions [12, 13, 38]. In addition, we applied the machine learning algorithm to both cortical and subcortical structures which is particularly important in PSP which also affects the cortex, basal ganglia, and dentate nucleus.

By assessing all the major brain regions relevant to the established pathological staging of PSP, we were able to validate the pipeline against the best current PSP pathology staging scheme [2], providing confidence in the robustness of the pipeline and additional insights into PSP tau pathology. We found a strong correspondence between our automated tau quantification and the standard, manual staging approach. We were able to demonstrate that TA density in cortical areas showed the strongest relation to PSP stage, which is consistent with the PSP staging system where TA density is the focus in cortical regions when grading severity. We went further by investigating which regions are the most informative towards PSP pathology staging, finding that the dentate nucleus, frontal, and occipital regions were more informative than basal ganglia nuclei. This is consistent with the known severe involvement of the basal ganglia nuclei from stage 2 onwards as per the described PSP staging [2]. As a result, the severity of pathology in the basal ganglia contributes less to distinguishing between higher PSP stages. The occipital region involvement corresponds to the PSP stage 4 and onwards, which likely explains the strong correlation between tau hallmark density in the occipital lobe and PSP stage.

To build on these insights, we investigated the relationship between measured tau type density and manually assessed region-specific severity rating to understand which tau types most influence the severity rating. We found that TA (and TF) density correlated strongest with the severity rating in the putamen amongst all the correlation assessed. Glial tau density also generally showed higher correlation to the severity rating than neuronal tau density. In subcortical regions, the low correlation between NFT density and severity rating could partly be due to the early occurrence hence saturation of neuronal tau inclusions in these regions. However, it is also important to consider the effect of neuronal depletion after tau deposition. The total number of neurons on each thin-sliced section of globus pallidus and subthalamic nucleus is low. It is possible that NFT formation and neuronal depletion creates an equilibrium state, resulting in NFT density being stable which contributed to the relatively low correlation with severity rating when compared to CB density as oligodendrocytes would be more abundant. These findings demonstrate the utility of automated quantitative neuropathology to validate and investigate the staging and progression of tau neuropathology.

Given our algorithm’s ability to quantify distinct types of tau inclusion, we investigated how the quantity and type of tau inclusions were related to clinical severity at the last point measured. We confirmed that the most advanced PSP neuropathology stage 6 had the most advanced clinical syndrome measured by PSPRS scores, and we identified that cortical tau density and subcortical NFT density were strongly associated with clinical severity measured by the PSPRS. We found largely insufficient evidence to demonstrate a linear relationship between tau burden and PSPRS score when quantified from all regions in the study. The use of Bayesian statistics indicated that more data would help to test (accept or reject) this association. The use of a Bayesian approach also enabled us to demonstrate that tau burden in subcortical regions in general is *not* associated with the PSPRS score; only when NFT burden is considered alone is there an association. Overall, these results highlight the importance in PSP of tau type-specific burden in specific anatomical locations, instead of simply investigating total tau burden in all regions.

There remain limitations to our study. We would have liked to compare between subtypes of PSP, but there were insufficient data from non-PSP-RS donor participants. Despite the high accuracy and robustness of the pipeline, it is designed to only classify tau pathologies that are specific to PSP. If the *postmortem* slide has coexisting tauopathies such as Ageing-related Tau Astrogliopathy, Primary Age-related tauopathy or Alzheimer-type neurofibrillary tangle, neuritic plaque and thread pathology, the pipeline may not yield accurate results because it has not ‘seen’ them before. These coexisting pathologies are not uncommon in PSP but are generally mild in severity so in most cases their impact is minimal [39–41]. However, we excluded 8 slides with significant co-pathology. In keeping with the PSP staging scheme, we did not assess other important brain regions involved in PSP such as the midbrain tegmentum, substantia nigra, thalamus, and brainstem regions. Moreover, as the pipeline relies on DAB thresholding to detect tau objects, iron granules may be included as tau objects. In this study, we manually removed iron granules, which was a time-consuming step and can be prone to error. Automating iron granule removal is challenging since they are heterogeneous across slides and affect some regions, such as the basal ganglia, more than others. The tau fragment class is made up of parts of axonal tau threads, tufted astrocyte processes and other tau fragments. This presents a challenge to a truly accurate quantification, since a large proportion of these fragments will be associated with larger tau inclusions. However, it is not possible to accurately assess this on a 2-dimensional neuropathology slide. Nevertheless, quantifying the density of these fragments appears to be useful in the assessment of tau stage and severity. Finally, this study included few early stage PSP donors (stage 2 or less), who are relatively rare in brain bank cohorts [42]. Nevertheless, we were able to observe an expected pattern of progression across stages from the current dataset.

## Conclusion

We have developed a highly accurate digital tau aggregate type-specific quantification for PSP *postmortem* brain which has also shown high correspondence the current consensus PSP staging system. We have shown the importance of studying tau aggregate type-specific burden in different brain regions as opposed to overall tau, to gain insights into the pathogenesis and progression of tauopathies. Having a reliable and robust automated quantification of tau pathology will catalyse future analysis to better understand the progression of tau pathology in PSP. We anticipate our approach can be adapted to other similar neurodegenerative tauopathies and proteinopathies. This will enable analysis of neuropathology at scale across brain regions and larger numbers of participants than is currently possible.

### Supplementary Information

**Additional file 1**. This file contains supplementary figures showing additional information on 1) examples of correct and incorrect tau classification, and 2) bayesian model results.

## Data Availability

The data that support the findings of this study are available from the brain bank network but restrictions apply to the availability of these data, which were used under license for the current study, and so are not publicly available. Data are however available from the authors upon reasonable request and with permission of the brain bank network. Code used in this study is available at: https://gitlab.developers.cam.ac.uk/tp500/digital-neuropathology.

## Abbreviations

BF: Bayes factor
CB: Coiled bodies
DAB: 3,3′-Diaminobenzidine
DN: Dentate nucleus
GP: Globus pallidus
H-DAB: Hematoxylin-DAB
IQR: Interquartile range
NERD: Neuropathology Research in Dementia
NFT: Neurofibrillary tangles
PR-AUC: Area under the precision-recall curve
PSP: Progressive Supranuclear Palsy
PSPRS: PSP rating scale
PU: Putamen
ROPE: Region of Practical Equivalence
STN: Subthalamic nucleus
TA: Tufted astrocytes
TF: Tau fragments
