# Supplementary material for: Accurate digital quantification of tau pathology in progressive supranuclear palsy
Source: Acta Neuropathol Commun. 2023 Nov 9;11:178. doi: 10.1186/s40478-023-01674-y (PMC10634011; doi:10.1186/s40478-023-01674-y)

Supplementary materials

“Accurate digital quantification of tau pathology in progressive supranuclear palsy” by Tanrada Pansuwan, Annelies Quaegebeur, Sanne S. Kaalund, Eric Hidari, Mayen Briggs, James B. Rowe, and Timothy Rittman

Extra examples of correct and incorrect classification

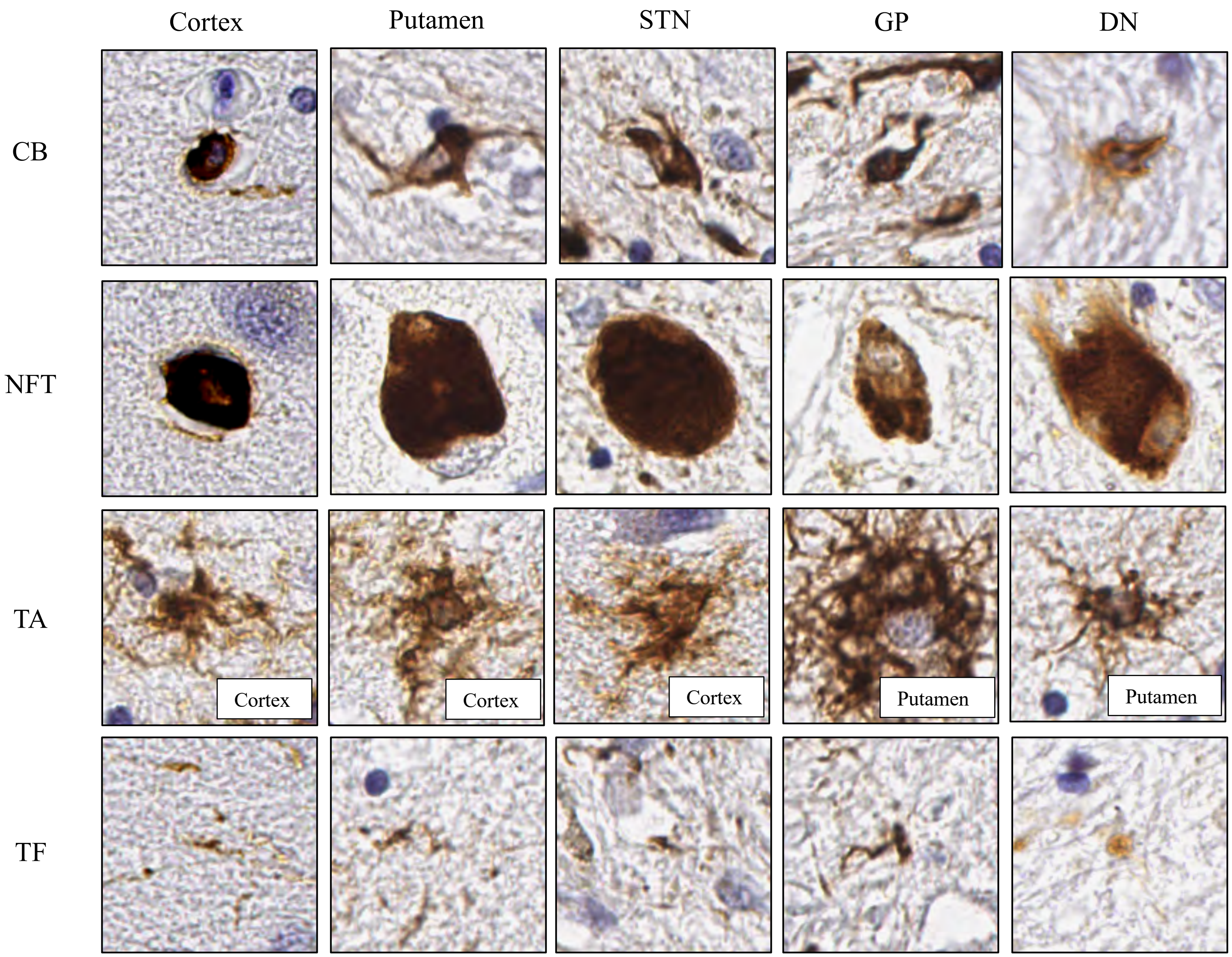

Figure 1: Examples of correct classification from the held-out test set for each tau aggregate type from the cortex, putamen, subthalamic nucleus (STN), globus pallidus (GP) and dentate nucleus (DN). All images were cropped 150 x 150 mm window size. TA examples are only drawn from the cortex and putamen. CB coiled body; NFT neurofibrillary tangle; TA tufted astrocyte; TF tau fragments.

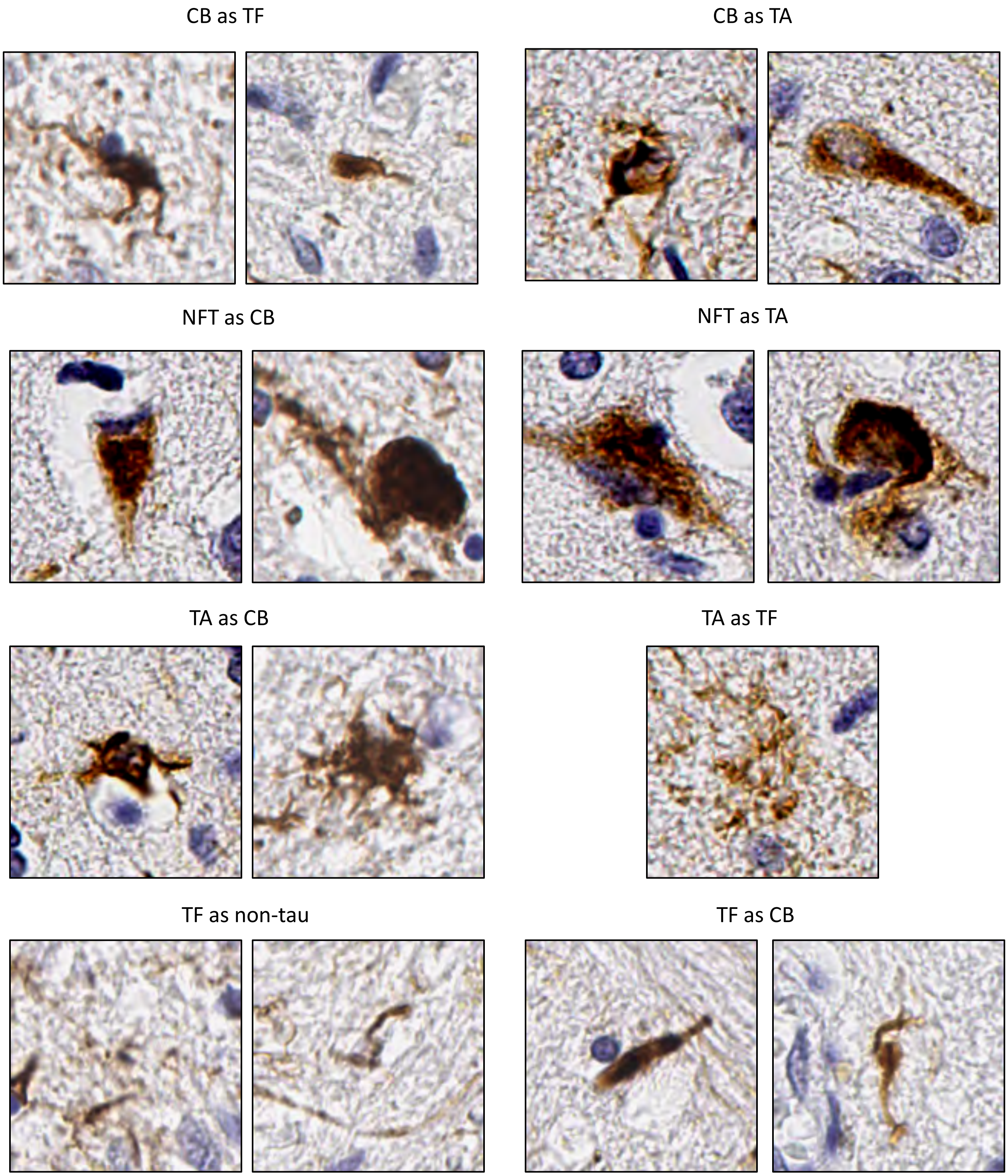

Figure 2: Example of possible misclassification observed between tau aggregate types from the held-out test set. Truth label as predicted label is presented. All images were cropped 150 x 150 mm window size. CB coiled body; NFT neurofibrillary tangle; TA tufted astrocyte; TF tau fragments.

Bayesian regression results

Final models only

PSPRS score ~ PSP stage + disease duration + PSPRS-death interval

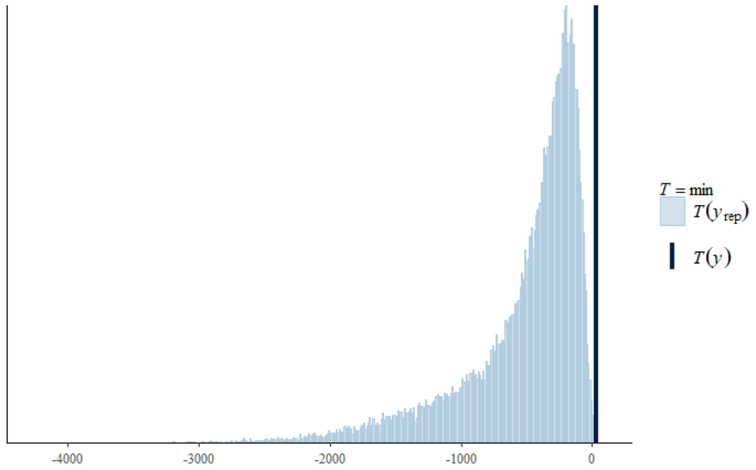

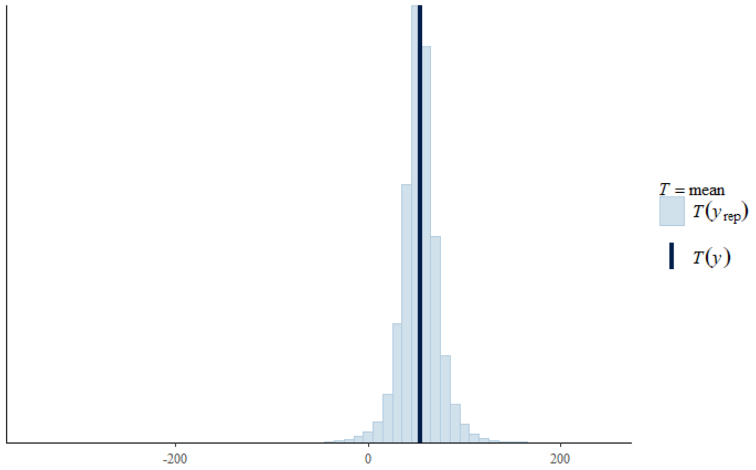

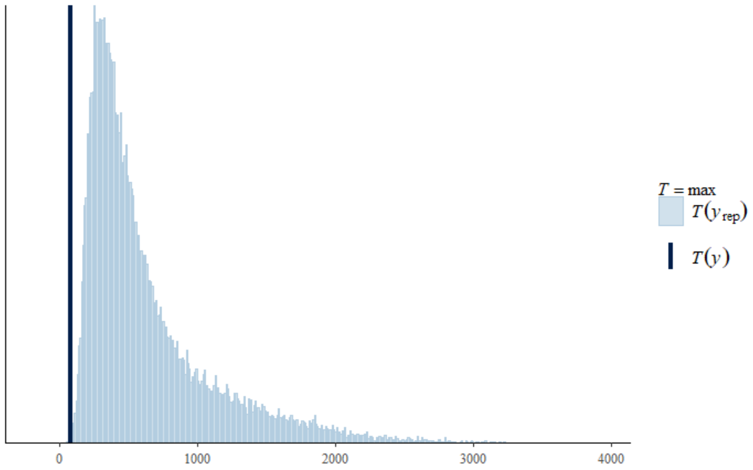

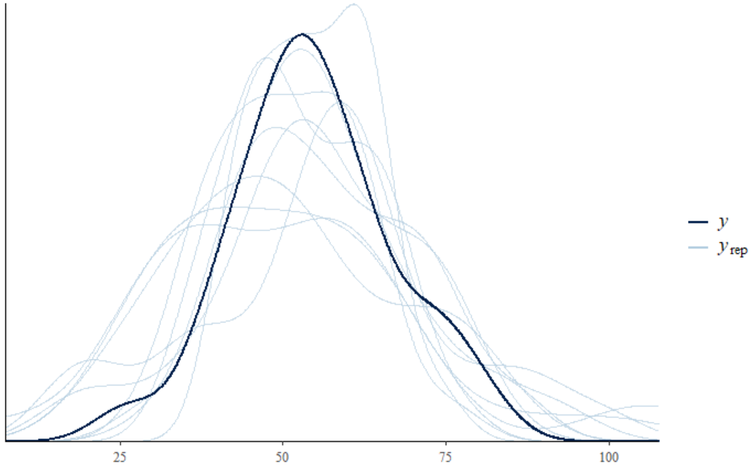


```
 Family: gaussian
  Links: mu = identity; sigma = identity
Formula: PSPRS.Total..last.assesment. ~ Stage + disease.duration..years. + years.PSPRS.and.death
   Data: data_PSPRS (Number of observations: 28)
  Draws: 4 chains, each with iter = 20000; warmup = 10000; thin = 1;
         total post-warmup draws = 40000

Population-Level Effects:
                           Estimate Est.Error l-95% CI u-95% CI Rhat Bulk_ESS Tail_ESS
Intercept                     35.84      9.04    18.01    53.74 1.00    21713    25208
Stage3                         5.40     11.08   -16.44    27.20 1.00    22308    26378
Stage4                         7.68      8.68    -9.36    24.83 1.00    18989    23866
Stage5                         9.96      8.80    -7.29    27.40 1.00    18571    23418
Stage6                        29.59     11.15     7.75    51.57 1.00    22480    25702
disease.duration..years.       1.36      0.73    -0.08     2.80 1.00    33214    27678
years.PSPRS.and.death         -0.27      3.16    -6.56     6.01 1.00    31659    27764

Family Specific Parameters:
      Estimate Est.Error l-95% CI u-95% CI Rhat Bulk_ESS Tail_ESS
sigma    11.07      1.78     8.23    15.20 1.00    24687    26906

Draws were sampled using sampling(NUTS). For each parameter, Bulk_ESS
and Tail_ESS are effective sample size measures, and Rhat is the potential
scale reduction factor on split chains (at convergence, Rhat = 1).
```

PSPRS score ~ PSP stage + disease duration + PSPRS-death interval

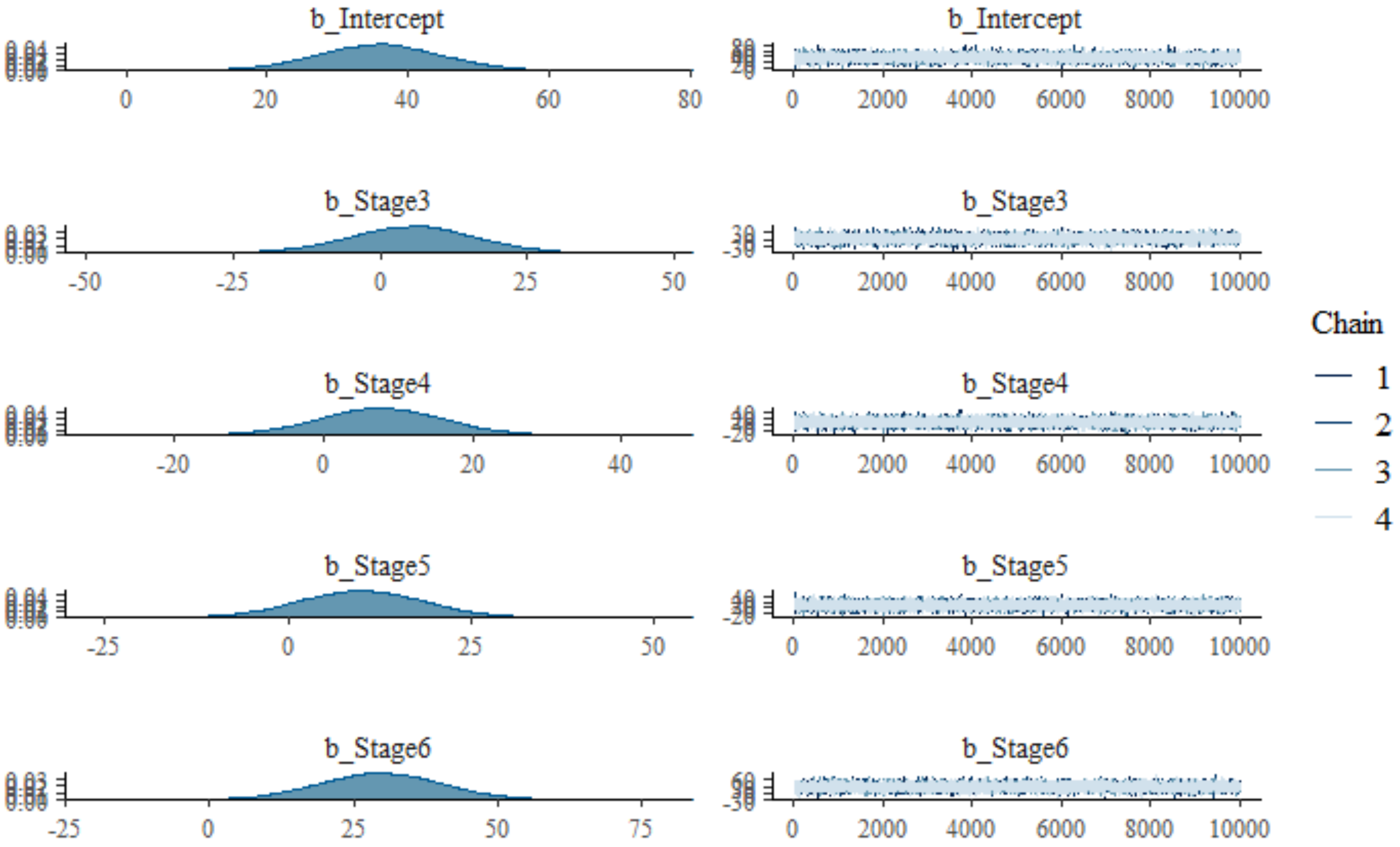

PSPRS score ~ PSP stage + disease duration + PSPRS-death interval

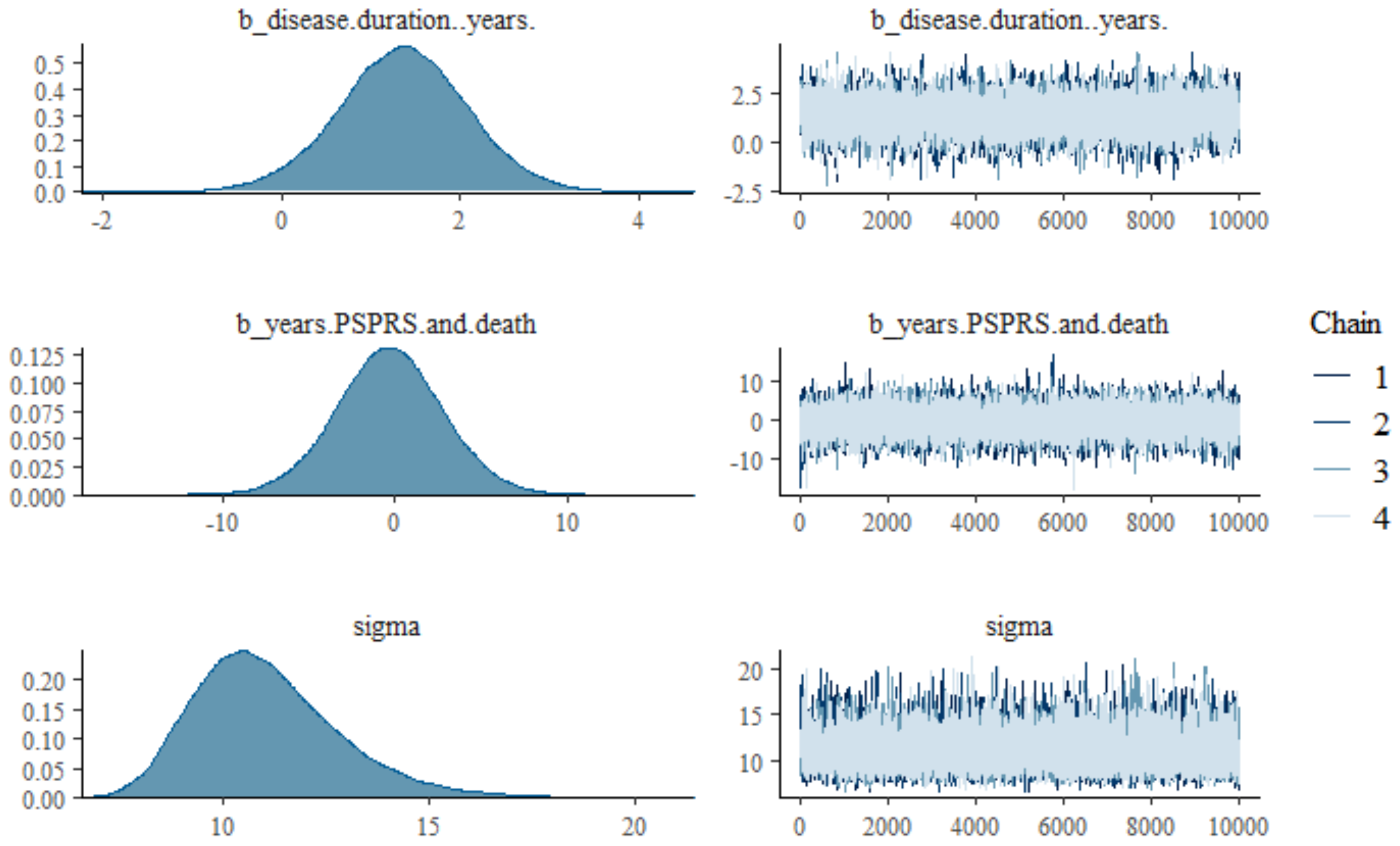

PSPRS score ~ total tau burden (all regions) + disease duration + PSPRS-death interval

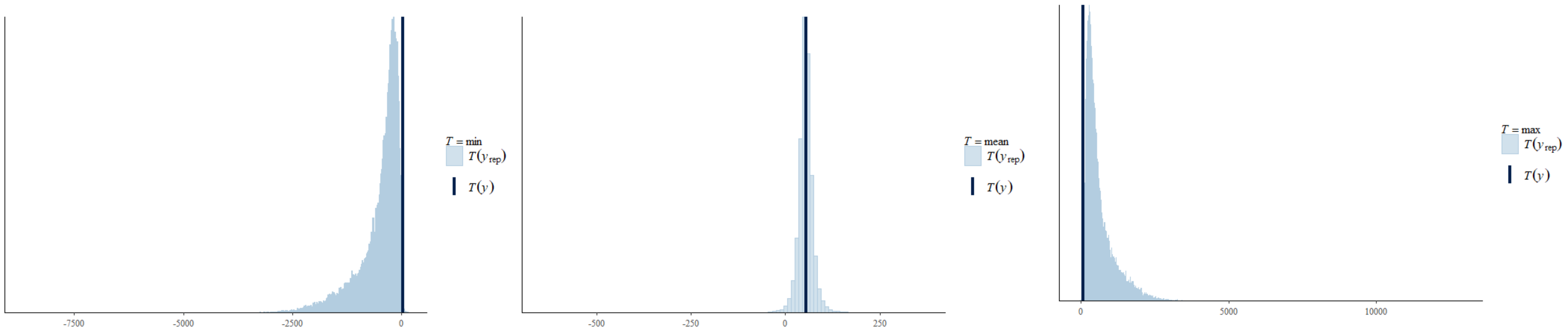

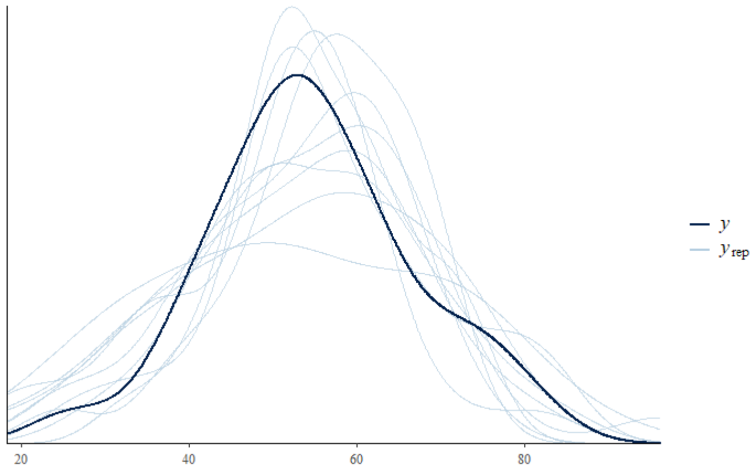


```
 Family: gaussian
  Links: mu = identity; sigma = identity
Formula: PSPRS.Total..last.assesment. ~ mean_total_tau_density_log + disease.duration..years. +
years.PSPRS.and.death
   Data: selected_dat (Number of observations: 28)
  Draws: 4 chains, each with iter = 20000; warmup = 10000; thin = 1;
         total post-warmup draws = 40000

Population-Level Effects:
                           Estimate Est.Error l-95% CI u-95% CI Rhat Bulk_ESS Tail_ESS
Intercept                     91.32     24.75    42.01   140.14 1.00    44635    29960
mean_total_tau_density_log    10.93      5.52    -0.07    21.83 1.00    43093    29770
disease.duration..years.       1.95      0.76     0.46     3.45 1.00    43340    29822
years.PSPRS.and.death         -1.81      2.97    -7.64     4.13 1.00    42616    30011

Family Specific Parameters:
      Estimate Est.Error l-95% CI u-95% CI Rhat Bulk_ESS Tail_ESS
sigma    11.40      1.70     8.64    15.27 1.00    34280    28932

Draws were sampled using sampling(NUTS). For each parameter, Bulk_ESS
and Tail_ESS are effective sample size measures, and Rhat is the potential
scale reduction factor on split chains (at convergence, Rhat = 1).
```

PSPRS score ~ total tau burden (all regions) + disease duration + PSPRS-death interval

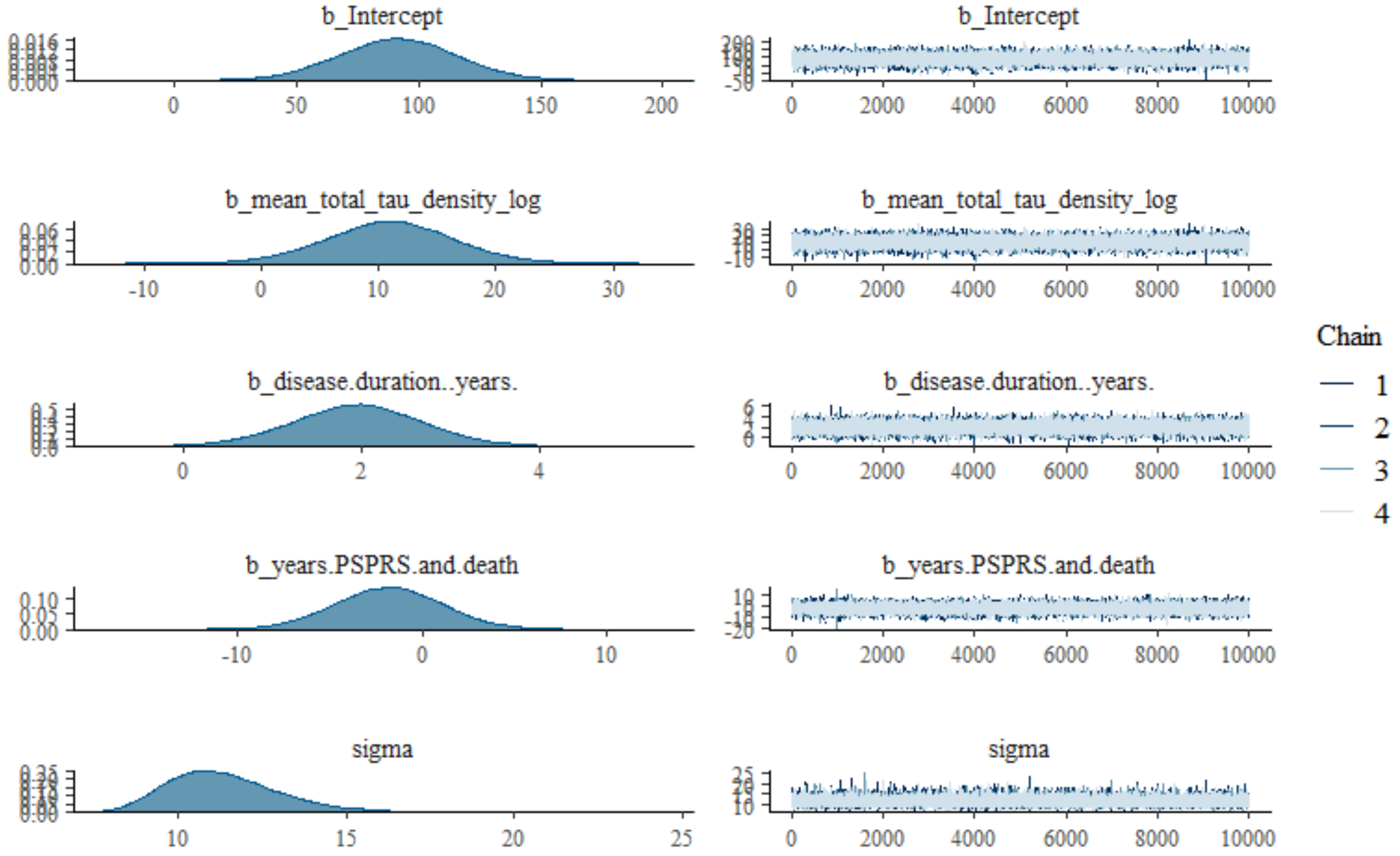

PSPRS score ~ total tau burden (cortical regions) + disease duration + PSPRS-death interval

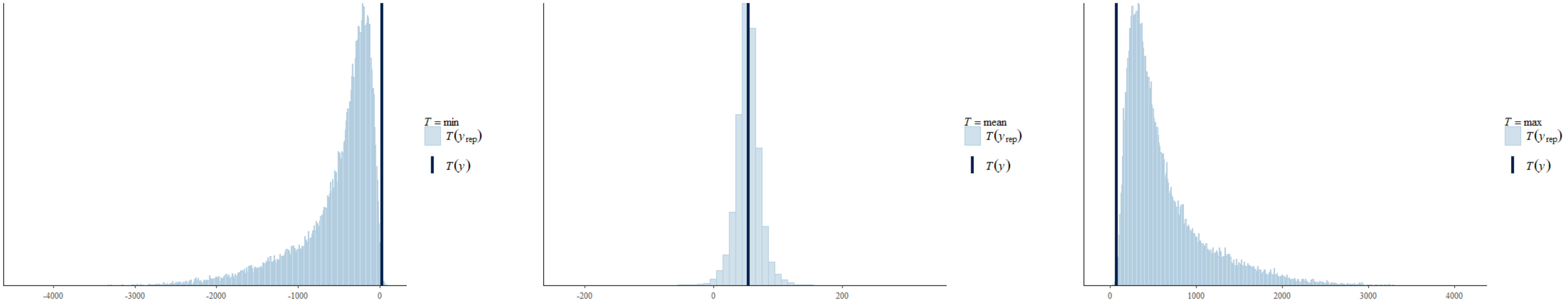

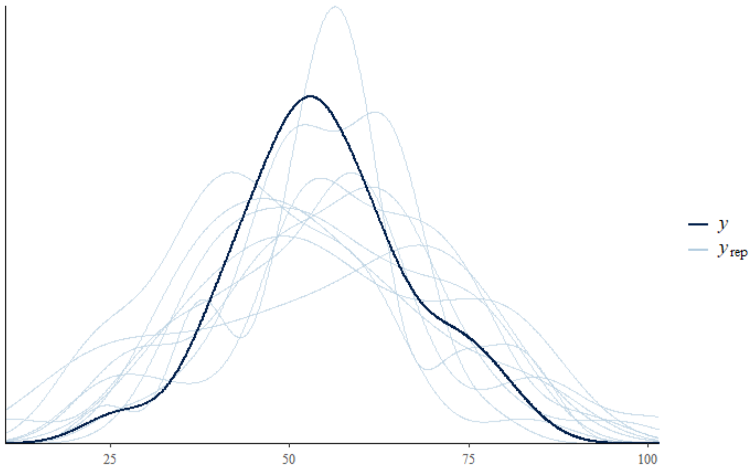


```
 Family: gaussian
  Links: mu = identity; sigma = identity
Formula: PSPRS.Total..last.assesment. ~ mean_total_tau_density_log + disease.duration..years. +
years.PSPRS.and.death
   Data: selected_dat (Number of observations: 28)
  Draws: 4 chains, each with iter = 20000; warmup = 10000; thin = 1;
         total post-warmup draws = 40000

Population-Level Effects:
                           Estimate Est.Error l-95% CI u-95% CI Rhat Bulk_ESS Tail_ESS
Intercept                     94.11     20.32    54.24   133.95 1.00    47918    27332
mean_total_tau_density_log    10.69      4.13     2.54    18.81 1.00    47938    28093
disease.duration..years.       1.61      0.71     0.20     3.03 1.00    46485    29611
years.PSPRS.and.death         -1.12      2.75    -6.53     4.31 1.00    49023    29347

Family Specific Parameters:
      Estimate Est.Error l-95% CI u-95% CI Rhat Bulk_ESS Tail_ESS
sigma    10.86      1.62     8.21    14.56 1.00    37419    29950

Draws were sampled using sampling(NUTS). For each parameter, Bulk_ESS
and Tail_ESS are effective sample size measures, and Rhat is the potential
scale reduction factor on split chains (at convergence, Rhat = 1).
```

PSPRS score ~ total tau burden (cortical regions) + disease duration + PSPRS-death interval

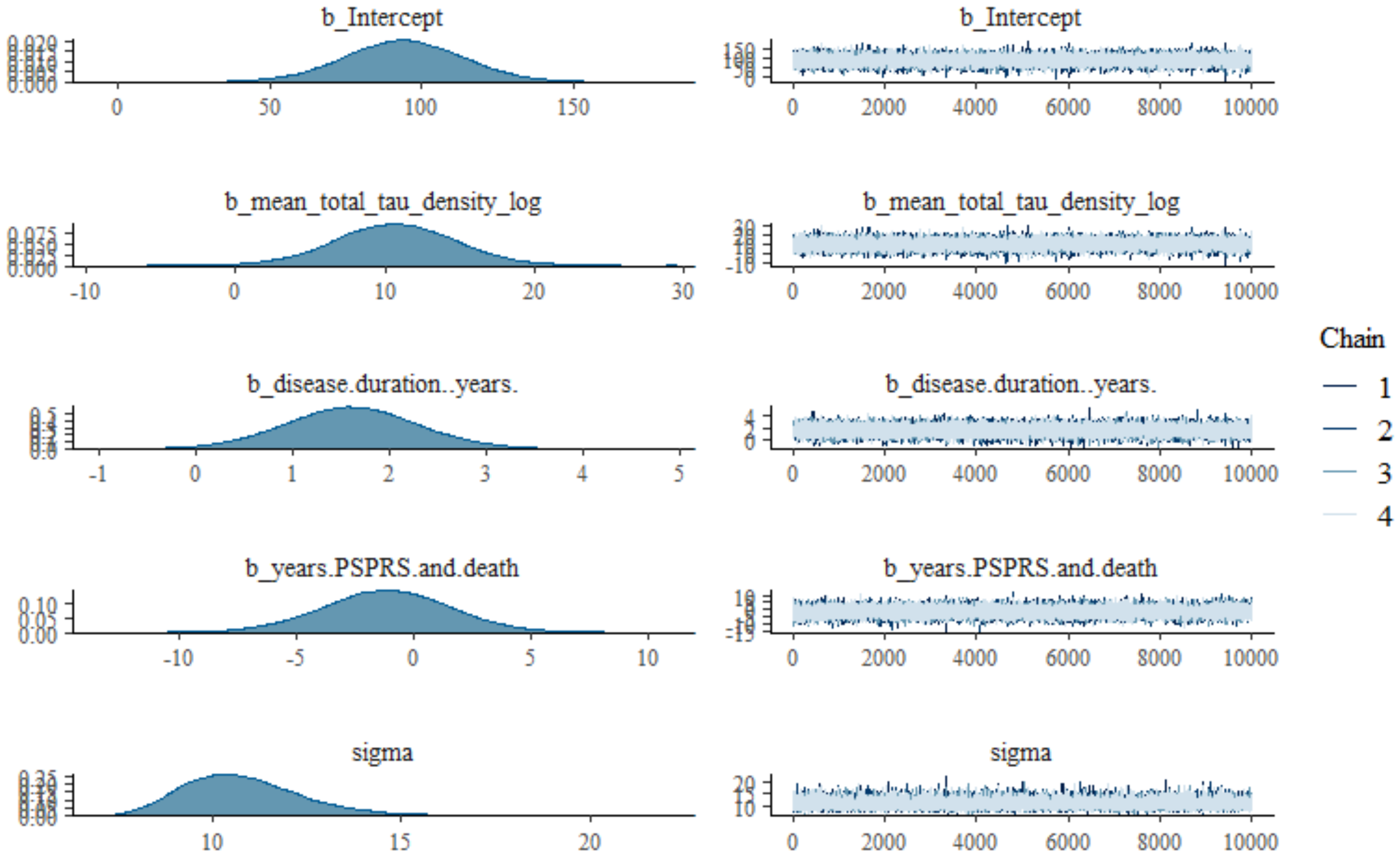

PSPRS score ~ NFT burden (subcortical regions) + disease duration + PSPRS-death interval

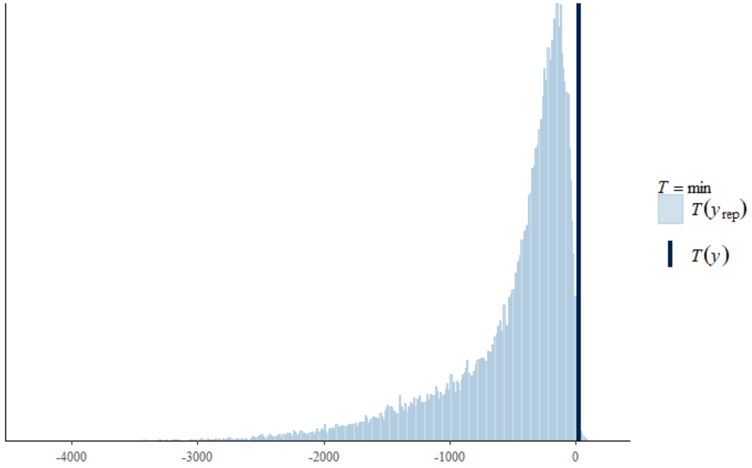

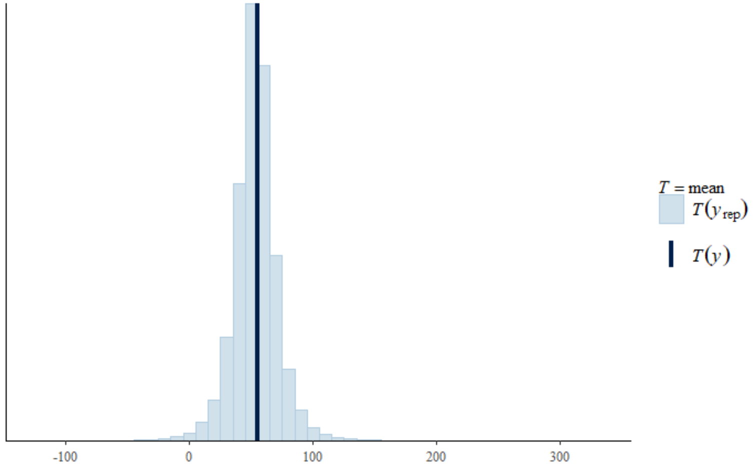

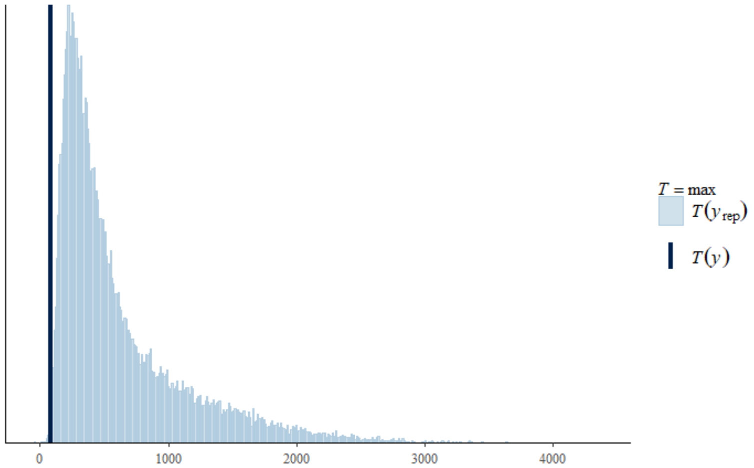

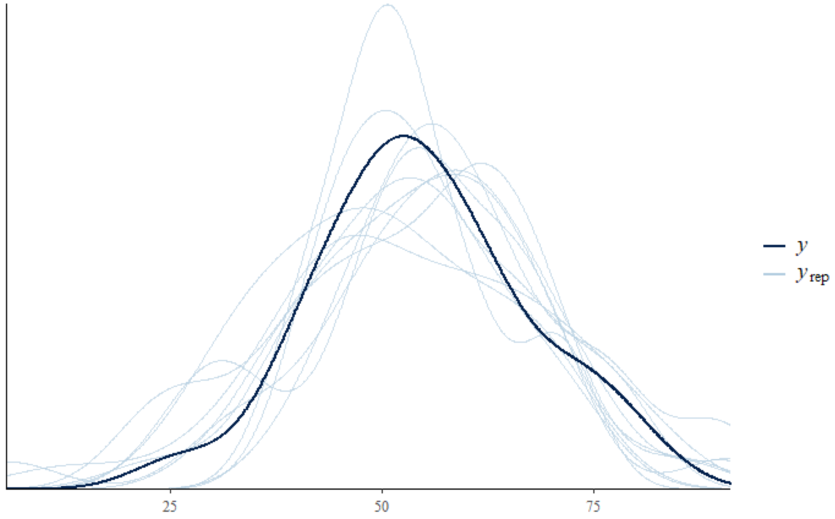


```
 Family: gaussian
  Links: mu = identity; sigma = identity
Formula: PSPRS.Total..last.assesment. ~ mean_NFT_density_log + disease.duration..years. + years.
PSPRS.and.death
   Data: selected_dat (Number of observations: 27)
  Draws: 4 chains, each with iter = 20000; warmup = 10000; thin = 1;
         total post-warmup draws = 40000

Population-Level Effects:
                         Estimate Est.Error l-95% CI u-95% CI Rhat Bulk_ESS Tail_ESS
Intercept                  129.06     40.55    48.86   208.81 1.00    42298    30783
mean_NFT_density_log        14.80      6.76     1.38    28.05 1.00    41054    30097
disease.duration..years.     2.74      0.85     1.05     4.43 1.00    41030    29428
years.PSPRS.and.death       -2.29      2.91    -8.07     3.46 1.00    41593    30086

Family Specific Parameters:
      Estimate Est.Error l-95% CI u-95% CI Rhat Bulk_ESS Tail_ESS
sigma    11.09      1.69     8.37    14.94 1.00    32610    28051

Draws were sampled using sampling(NUTS). For each parameter, Bulk_ESS
and Tail_ESS are effective sample size measures, and Rhat is the potential
scale reduction factor on split chains (at convergence, Rhat = 1).
```

PSPRS score ~ NFT burden (subcortical regions) + disease duration + PSPRS-death interval

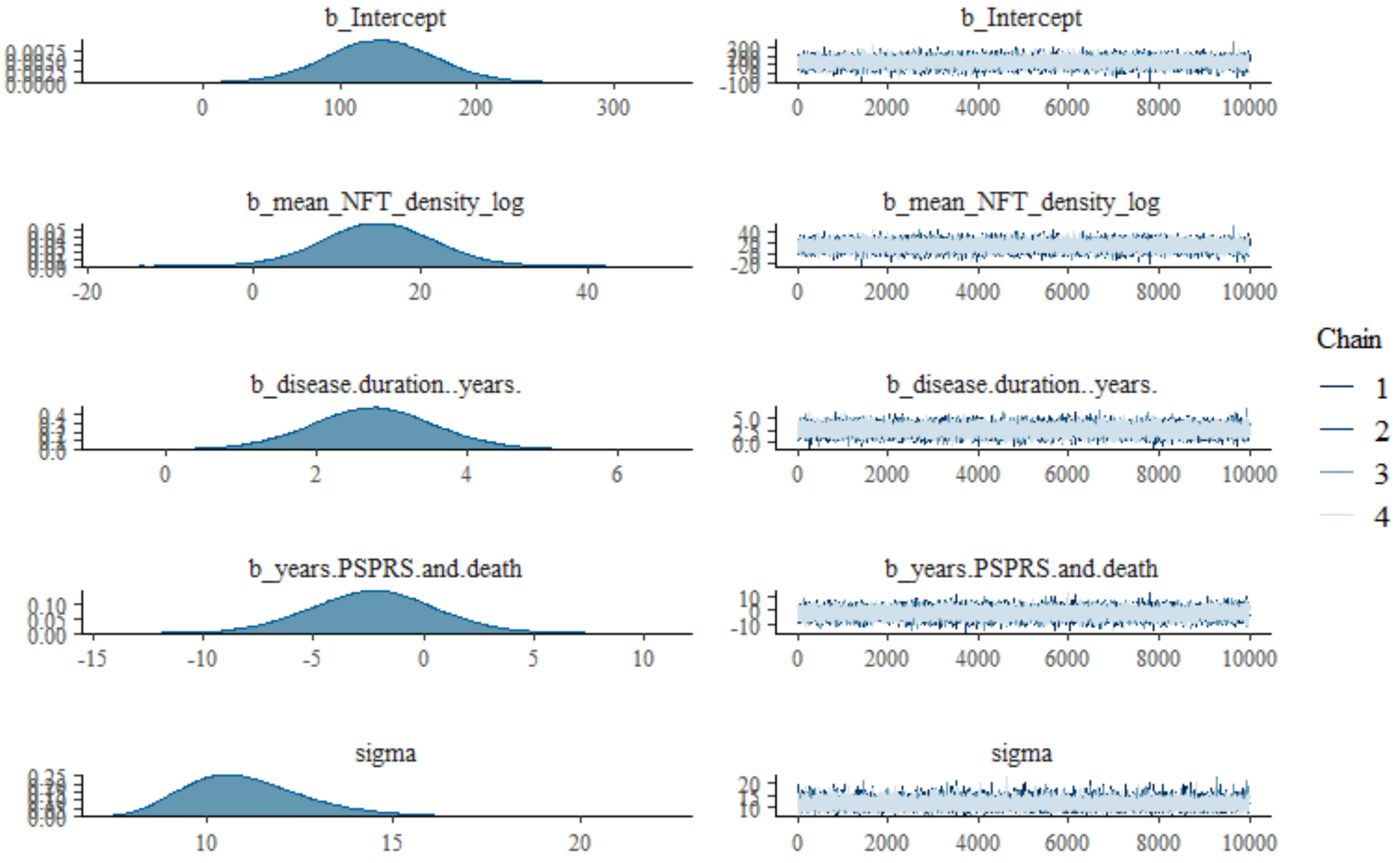

Supplement: Supplementary file 1 — Additional file 1. This file contains supplementary figures showing additional information on 1) examples of correct and incorrect tau classification, and 2) bayesian model results. [file 40478_2023_1674_MOESM1_ESM.pdf]
